# LncRNAs harbouring regulatory motifs within repeat elements modulate immune response towards COVID‐19 disease severity and clinical outcomes

**DOI:** 10.1002/ctm2.932

**Published:** 2022-07-08

**Authors:** Partha Chattopadhyay, Pallavi Mishra, Kriti Khare, Aanchal Yadav, Priyanka Mehta, Sheeba Saifi, Aparna Swaminathan, Priti Devi, Shaista Parveen, Akansha Tyagi, Vinita Jha, Bansidhar Tarai, Sujeet Jha, Sandeep Budhiraja, Jitendra Narayan, Rajesh Pandey

**Affiliations:** ^1^ INtegrative GENomics of HOst‐PathogEn (INGEN‐HOPE) Laboratory CSIR‐Institute of Genomics and Integrative Biology (CSIR‐IGIB) Delhi India; ^2^ Academy of Scientific and Innovative Research (AcSIR) Ghaziabad India; ^3^ Max Super Speciality Hospital (A Unit of Devki Devi Foundation) Max Healthcare Delhi India

To the Editor:

Modulators of COVID‐19 differential disease severity and clinical outcome deserve focused attention. In the background of paucity of comprehensive elucidation of the functional role of lncRNAs in COVID‐19 clinical subphenotypes (mild, moderate, severe and mortality), albeit infected by the same pathogen ‐ SARS‐CoV‐2, we undertook this novel study in a hospital admitted cohort of 117 patients in India. Our integrative analysis highlights important role of lncRNAs in regulating immune response with plausible functional role of transcription factor binding sites (TFBS) within the repeat elements of the significant differentially expressed (DE) lncRNAs.

Hospitalised COVID‐19 patients were stratified into subphenotypes based on their distinct disease phenotypes and outcomes as per Indian Council of Medical Research (ICMR). Patients outside the ICMR guidelines were stratified into two groups: respiratory support (RS) and shortness of breath (SOB) (detailed methodology, results and literature supporting data interpretation as File S1). Figure 1A summarises study highlighting patient segregation into subphenotypes, experimental methodologies and downstream analysis for lncRNA differential expression, lncRNA–miRNA–mRNA interaction, pathway enrichment and role of regulatory sites with the repeat elements.

**FIGURE 1:**
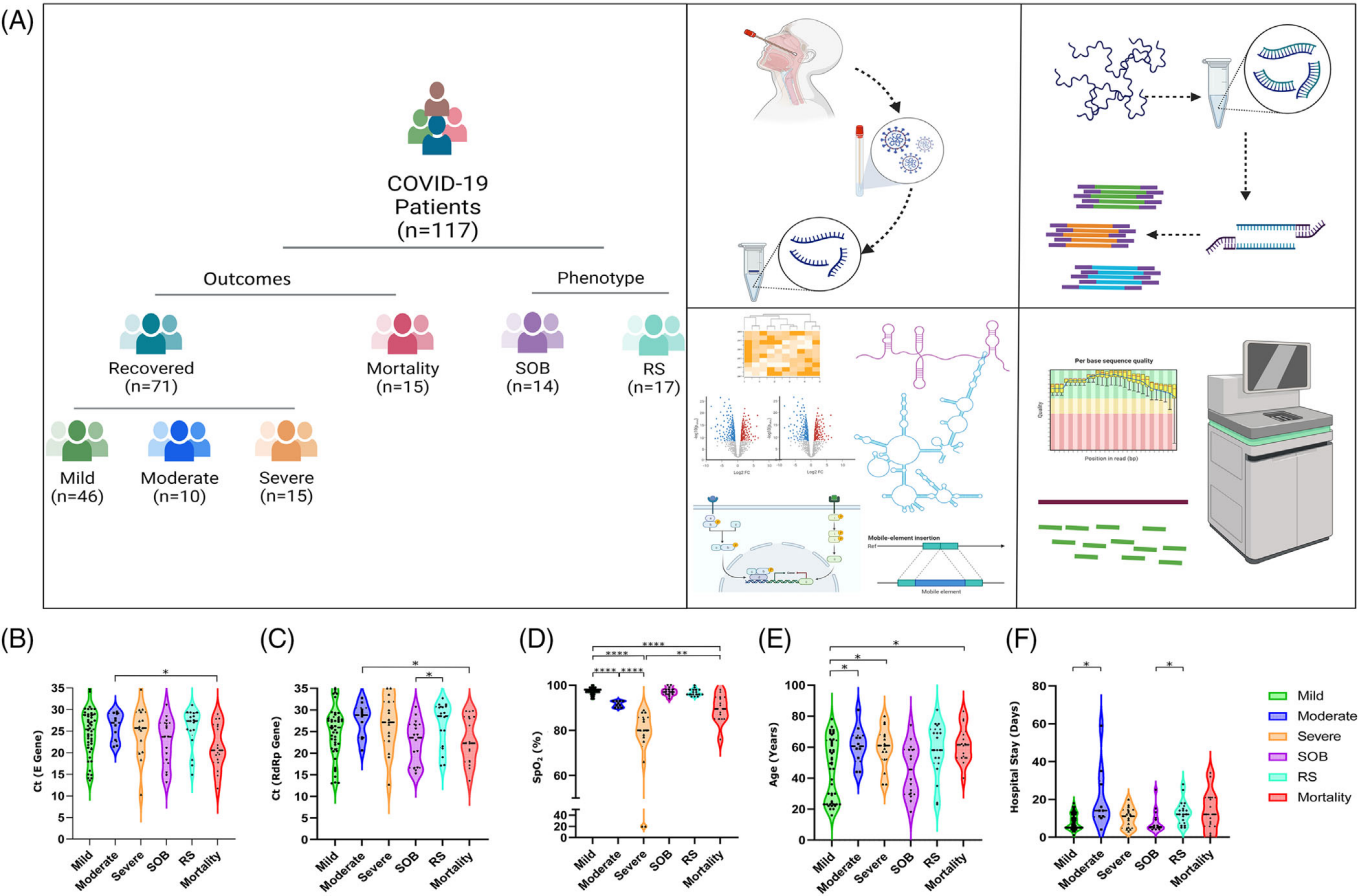
Overview of study design, patient segregation with clinical characterisation and significant analysis. (A) Sample distribution and schematic workflow for transcriptomic analysis, followed by analysis for differentially expressed lncRNAs, downstream functional analysis and visualisation. (B–F) Sample‐wise distribution of clinical parameters across subphenotypes along with their statistical significance. (B) *C*_t_ value of E gene, (C) *C*_t_ value of RdRp gene, (D) SpO_2_ level, (E) age in years, and (F) hospital stay in days. *p‐value < .05, **p‐value < .01, ***p‐value < .001, ****p‐value < .0001

The patient demographics and clinical data are summarised in File S1: Table S1, wherein, the median *C*_t_ value of *E/RdRp* gene was significantly different between recovered/mortality and RS/SOB patients, respectively (Figure 1B,C). SpO_2_ level was significantly different between recovered/mortality and mild/moderate/severe patients (Figure 1D), in addition to median age being different between mild/moderate/severe and mortality patients (Figure 1E). The duration of hospital stay was also significantly different in mild/moderate/severe and RS/SOB categories (Figure 1F).

To understand role of lncRNAs in modulating host response in the patients who succumbed to COVID‐19, we performed differential expression analysis between recovered and mortality patients. We found three lncRNAs significantly downregulated in the *mortality patients* (File S2; Figure 2A), with Figure 2B highlighting top 20 DE lncRNAs. Integration of lncRNA–miRNA–mRNA regulatory potential revealed that by virtue of LINC00174:11 downregulation in mortality, miR‐1910‐3p‐mediated elevation of NF‐kB signalling and cytokine storm were possible.^1^ Downregulation of RNASEH1‐AS1:23 and ROR1‐AS1:6 may modulate heightened immune, inflammatory and stress response, as well as viral replication during mortality, mediated by miR‐218‐5p and miR‐375.^2^, ^3^ DEG and GSEA analysis of study cohort in conjunction with LncRNA–miRNA–mRNA interaction network, highlight heightened inflammatory response (Files S3–S5; Figure 2C,D).

**FIGURE 2:**
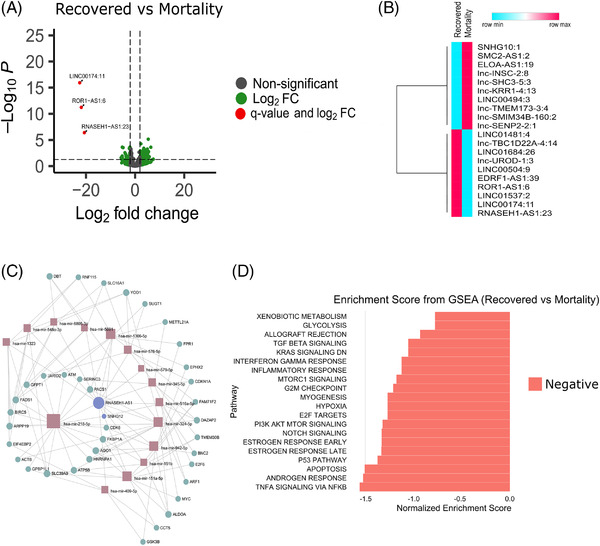
Differentially expressed lncRNAs in the recovered patients (compared to mortality) and functional analysis. (A) Volcano plot showing the differential expression of lncRNAs in recovered versus mortality patients. The green dots represent lncRNAs with more than log2 fold change of ±2 only, while red dots represent lncRNAs significant based on log2 fold change and *q*‐value both. (B) Differential expression profile of top 20 lncRNAs (based on log2 fold change) based on average normalised counts between recovered and mortality patients. (C) lncRNA–miRNA–mRNA interaction network, brown box represents miRNA, purple circle represents lncRNAs and green circle represents mRNAs. (D) Gene set enrichment analysis of the genes interacting with the differentially expressed lncRNAs, where *x*‐axis represents the normalised enrichment score (NES), and the colour represents the direction of NES

Subsequently, to elucidate the role of lncRNAs in modulating host response to COVID‐19 disease trajectories, we identified DE lncRNAs among COVID‐19 subphenotypes (mild vs. moderate/severe/mortality, moderate vs. severe/mortality and severe vs. mortality) (Figure 3A,B; File S2). We observed LINC00294:1 upregulation and LINC00504:9 and RNASEH1‐AS1:23 downregulation‐mediated decreased inflammatory responses in the moderate (vs. mild), whereas MALAT1 downregulation‐indicated heightened immune response in moderate patients.^4^ The downregulation of UGDH‐AS1:11 in the severe indicates a MOV10 and UPF1‐mediated decreased antiviral response.^5^ Downregulation of LINC00504:9 also indicates decreased immune response in the severe patients. In the mild versus mortality patients, we observed downregulation of MALAT1:9, LINC00504:9 and RNASEH1‐AS1:23 in the mortality. Downregulation of LINC00504:9 and RNASEH1‐AS1:23 suggests a decreased inflammatory and antiviral response in the mortality patients, whereas downregulation of MALAT1:9 suggests an increased innate immune response in the mortality, contrary to other findings.^6^ Upregulation of LUCAT1:3 in mortality indicates activation of interferon immunity, whereas downregulated LINC01537 reflects increased iNOS‐mediated stress and decreased T‐cell activation in mortality.^7^, ^8^ MALAT1:9 upregulation in the severe (vs. moderate) indicates decreased immune response in severe, whereas UGDH‐AS1:11 downregulation suggests decreased antiviral response and increased disease severity in the severe.^5^ Finally, LINC00273 downregulation in the mortality group (vs. severe) could possibly explain the decreased early innate immune response in mortality.

**FIGURE 3:**
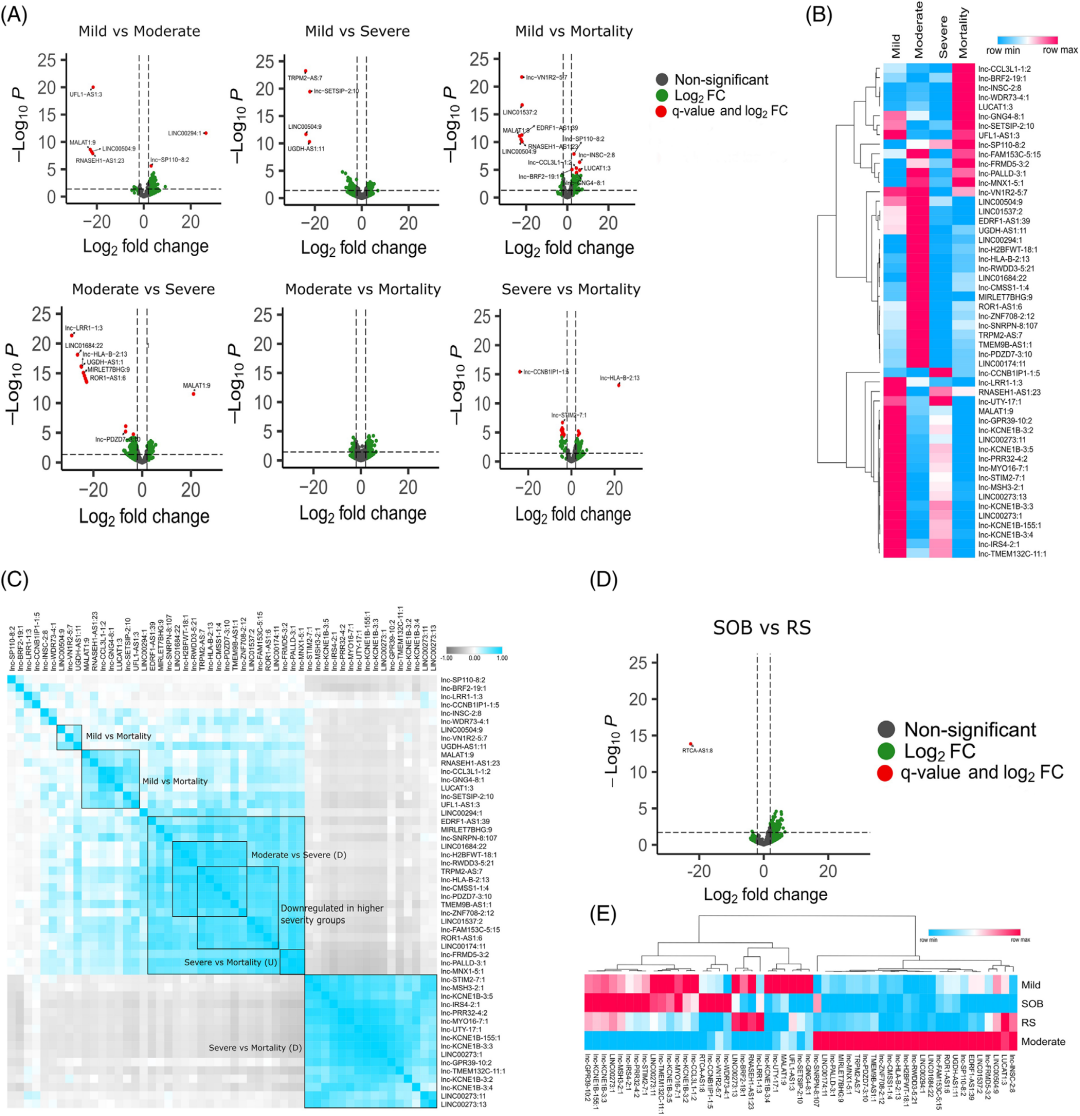
Differential expression of lncRNAs across COVID‐19 subphenotypes. (A) Volcano plot showing the differential expression of lncRNAs in the COVID‐19 subphenotypes. The green dots represent lncRNAs with more than log2 fold change of ±2 only; red dots represent significant lncRNAs based on both log2 fold change and *q*‐value. (B) Expression profile of the differentially expressed lncRNAs across groups (based on average normalised counts). (C) Pearson correlation plot of the differentially expressed lncRNAs across groups. The clusters are highlighted, where the expression of the lncRNAs is mentioned by U (upregulated) or D (downregulated). All the positively correlated clusters are statistically significant (*p* ≤ .05). (D) Volcano plot showing the differential expression of lncRNAs between RS and SOB groups. (E) Expression profile of the differentially expressed lncRNAs across mild, moderate, SOB and RS (based on average normalised counts)

Pearson correlation analysis of DE lncRNAs across subphenotypes revealed distinct lncRNA expression pattern correlating with disease severity (Figure 3C). Large cluster of 16 lncRNAs (lnc‐STIM2‐7:1, lnc‐MSH3‐2:1, lnc‐KCNE1B‐3:5, lnc‐IRS4‐2:1, lnc‐PRR32‐4:2, lnc‐MYO16‐7:1, lnc‐UTY‐17:1, lnc‐KCNE1B‐155:1, lnc‐KCNE1B‐3:3, LINC00273:1, lnc‐GPR39‐10:2, lnc‐TMEM132C‐11:1, lnc‐KCNE1B‐3:2, lnc‐KCNE1B‐3:4, LINC00273:11, LINC00273:13) was obtained downregulated in mortality patients, suggesting possible association of these lncRNAs with COVID‐19 mortality. We found an autophagy‐related antisense transcript RTCA‐AS1:8 to be downregulated in the RS compared to the SOB patients (Figure 3D). Importantly, downregulation of the RTCA‐AS1 in SARS‐CoV‐2‐infected human bronchial organoids has been reported.^9^ Additionally, we found that SOB patients were closer to the mild, while the RS were similar to moderate for their lncRNA expression (Figure 3E).

LncRNA–miRNA–mRNA interaction network and DEG analysis helped us to understand the possible biological functions of the DE lncRNAs (Figure S2A–E), followed by GSEA for the interacting genes (Files S3–S5; Figure S2F–M). We found lncRNA‐mediated regulation of CALM3/VAV3/WIPI2/MAD2L1/CDKN1A/CD47/IGF1R/ACTB genes, leading to immune/inflammatory response and some housekeeping biological function regulation.

For understanding the possible mechanism of gene regulation by specific lncRNAs, we analysed for repeat element distribution within the DE lncRNAs, with focus on LINE and SINE repeat elements (File S6). We observed significantly higher distribution of SINE/Alu, SINE/MIR and LINE/L1 elements across the comparison groups (Figure 4A,B). The distribution of SINE/Alu and LINE/L1 was higher in our DE lncRNA compared to that of overall distribution of these repeats (File S6; Figure S3A,B). Importantly, higher presence of Alu elements (SINE) was found in the DE lncRNAs in the mortality patients. This indicates heightened stress response during mortality as highlighted by existing literature, suggesting functional role of Alu repeats during viral infection.^10^ Subsequently, we analysed for the genes present within 5 kb upstream and downstream of the seven lncRNAs (12 genes) and performed pathway enrichment analysis to understand the biological functions of the genes (File S7; Figure 4C). Based on the pathway enrichment analysis, we selected TRPM2‐AS1 and RNASEH1‐AS1, and explored the role of TFBS in regulating the overlapping genes. We found four TFs (majorly bind to TFBS within Alu and L1 elements in TRPM2‐AS1), SOX2, GATA3, FOXO1 and FOXO3, to regulate the TRPM2 gene expression, while TFs, SOX10 and GATA6, bind with RNASEH1‐AS1 to regulate the RPS7 expression (Figure 4D). This highlights possible TFBS‐mediated regulation of genes upstream/downstream of the lncRNAs.

**FIGURE 4:**
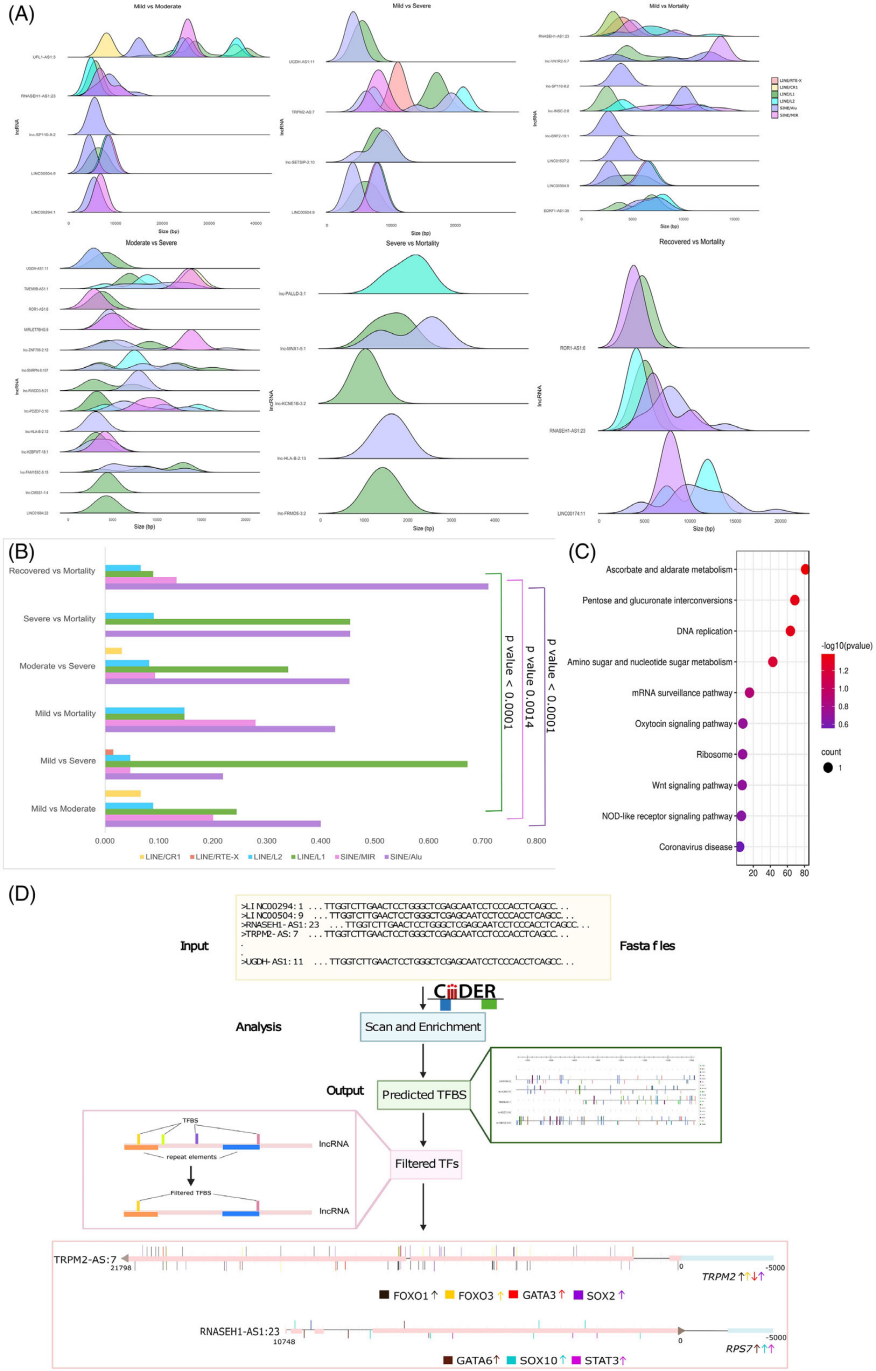
Repeat element distribution and functional analysis of TFBS within lncRNAs. (A) Density plot of the LINEs/SINEs within lncRNAs (frequency of repeat elements normalised to the length of the LncRNA), where the *x*‐axis represents the length of the lncRNAs. (B) Frequency distribution of the repeat elements across COVID‐19 subphenotypes. (C) Pathway enrichment analysis of the genes present within ±5 kb upstream and downstream of the lncRNAs, having LINES/SINEs within them. The *x*‐axis represents the enrichment score, circle size the number of genes involved in the pathway and the colour of the circle the significance of the pathway. (D) TFBS prediction within the repeat element regions in lncRNAs, and transcription factor‐mediated regulation of genes overlapping with lncRNAs. The arrow represents the transcription factor‐mediated upregulation/downregulation of the overlapping genes

In summary, our study highlights lncRNA‐mediated dysregulation of immune and stress responses and their potential mechanism during the early phase of SARS‐CoV‐2 infection, which potentially modulates different degrees of disease severity subphenotypes: mild, moderate, severe and mortality.

## CONFLICT OF INTEREST

The authors declare that there is no conflict of interest.

## FUNDING INFORMATION

Bill and Melinda Gates Foundation, Grant Number: INV‐033578

## Supporting information

FileS1

FileS2

FileS3

FileS4

FileS5

FileS6

FileS7

FigureS1

FigureS2

FigureS3
