# Supplementary material for: LncRNAs harbouring regulatory motifs within repeat elements modulate immune response towards COVID‐19 disease severity and clinical outcomes
Source: Clin Transl Med. 2022 Jul 8;12(7):e932. doi: 10.1002/ctm2.932 (PMC9270577; doi:10.1002/ctm2.932)
Supplement: Supplementary file 1 — FileS1 [file CTM2-12-0-s008.docx]

**LncRNAs Harbouring Regulatory Motifs Within Repeat Elements Modulate Immune Response Towards COVID-19 Disease Severity and Clinical Outcomes**

**Running title:***LncRNA regulates immune responses in COVID-19 sub-phenotypes*

Partha Chattopadhyay^1,2,#^, Pallavi Mishra^1,#^, Kriti Khare^1,2,$^, Aanchal Yadav^1,2,$^, Priyanka Mehta^1,$^, Sheeba Saifi^1^, Aparna Swaminathan^1^, Priti Devi^1,2,^, Shaista Parveen^1^, Akansha Tyagi^3^, Vinita Jha^3^, Bansidhar Tarai^3^, Sujeet Jha^3^, Sandeep Budhiraja^3^, Jitendra Narayan^1,*^, Rajesh Pandey^1,2,*^

^1^INtegrative GENomics of HOst-PathogEn (INGEN-HOPE) laboratory, CSIR-Institute of Genomics and Integrative Biology (CSIR-IGIB), Mall Road, Delhi-110007, India.

^2^Academy of Scientific and Innovative Research (AcSIR), Ghaziabad-201002, India.

^3^Max Super Speciality Hospital (A Unit of Devki Devi Foundation), Max Healthcare, Delhi 110017, India.

^#^Joint First Authors, ^$^Joint Second Authors, ^*^Co-corresponding authors

**Contact Details**

Rajesh Pandey, PhD

Principal Scientist,

INtegrative GENomics of HOst-PathogEn (INGEN-HOPE) laboratory,

CSIR-Institute of Genomics and Integrative Biology (CSIR-IGIB),

North Campus, Near Jubilee Hall, Mall Road, Delhi-110007, India.

Email: rajeshp@igib.in; Tel.: +91 9811029551

**Graphical Presentation**

**
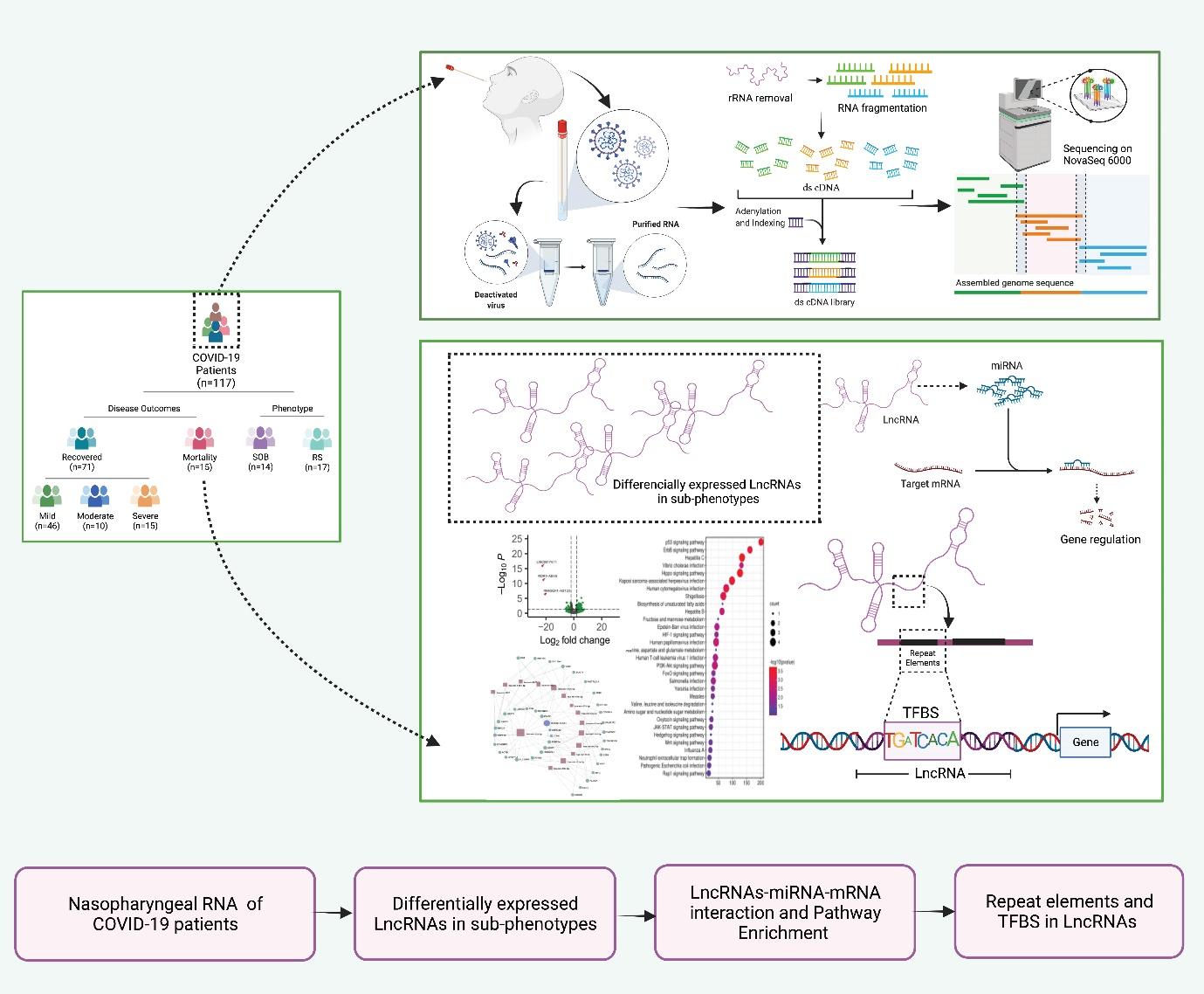
**

**Materials and Methods**

**Patient cohort, sampling and data collection**

*Sample Collection and Preprocessing*

The patients were admitted to tertiary care centre (MAX Super Speciality Hospital) in Delhi, India with confirmed COVID-19 positive status based on qRT-PCR results. Both nasopharyngeal/oropharyngeal swabs for each patient were collected in VTM by the paramedical staff at the hospital for qRT-PCR on the day of admission. Viral RNA from VTM was isolated using QIAmp viral mini kit, (Qiagen, Cat. No. 52906) and SARS-CoV-2 detection and quantification was performed using TRUPCR SARS-CoV-2 kit (3B BlackBio Biotech India Ltd., Cat. No. 3B304), with a cycle threshold of 35.

*Clinical Sub-phenotypes of Study Participants*

The patients were stratified into two broad groups based on the outcomes: Recovered and Mortality. The recovered group was further subdivided into three sub-phenotypes based on disease severity: Mild, Moderate, and Severe, as per Indian Council of Medical Research (ICMR) guidelines (Comprehensive Guidelines for Management of COVID-19 patients, Directorate General of Health Services, MoHFW, GOI). Briefly, SpO_2_ levels, requirement of respiratory support and/or breathlessness parameters were taken into consideration. In mild cases, the SpO_2_ level was ≥ 94% with no breathing problem. Moderate cases were defined as showing breathing difficulty with SpO_2_ levels ranging between 91-93%. Severe cases showed respiratory distress with respiratory support requirement and SpO_2_ levels < 90%. Mortality group was defined as patients who succumbed to COVID-19 during hospital stay. However, few patients reported shortness of breath and/or required respiratory support, despite having SpO_2_ level of >94%. Patients who required respiratory support despite >94% SpO_2_ level were categorized under RS (respiratory support). Patients (SpO_2_ >94%) who reported shortness of breath but did not require respiratory support were categorized as SOB (shortness of breath). All study procedures were in accordance with the Declaration of Helsinki and approved by the Institutional Ethics Committee of CSIR-Institute of Genomics and Integrative Biology and MAX Super Speciality Hospital. Informed consent was obtained from all individuals or their legal guardians.

**Library preparation and Sequencing**

A total of 250ng of total RNA isolated from the nasopharyngeal/oropharyngeal swabs were taken for sequencing library preparation using Illumina TruSeq® Stranded Total RNA Library Prep Gold (cat. no 20020599) as per manufacturer’s reference guide (1000000040499 v00) and our previous study [1]. Briefly, cytoplasmic and mitochondrial rRNA was removed using biotinylated target specific oligos with Ribo-Zero rRNA removal beads. The purified RNA was fragmented using divalent cations under elevated temperature. The cDNA synthesis includes, first strand cDNA was prepared from the cleaved RNA fragments using reverse transcriptase and random primers, followed by second strand synthesis using DNA polymerase 1 and RNase H. The blunt 3’ end of the double stranded cDNA was adenylated, followed by addition of indexes and final amplification to enrich the library. The final library was purified using AMPure XP (Beckman Coulter, A63881). Agilent 2100 bioanalyzer was used to check the library quality, followed by denaturation using 0.2N NaOH and sequencing on NovaSeq 6000, using NovaSeq S2 v1.5 reagents at 2×101 read length and loading concentration of 400pM.

**Quality Control, Mapping to Reference and Identification of DE-lncRNAs**

The schematic representation of the lncRNA DE-analysis strategy is depicted in **Supplementary Figure S1**. The core idea is to do statistical testing separately for each transcript We used R/Bioconductor packages DESeq2 for finding differential expression in RNA-seq data and applied it to transcript-level count data using the default parameters and following the directions in the package manuals to estimate transcript-level p-values [2].

FastQC is utilised in this pipeline to do quality control on the raw FASTQ files. Trimming is done with the Trimmomatic tool, and an extra QC report is provided [3]. To quantify read abundance or transcript expression levels, reads are mapped to the human reference transcriptome (GRCh38.p13) using Salmon quasi mapping tool , which generates a transcript quantification table. This pipeline also employs the HISAT2 aligner, which has relatively low memory needs [4, 5]. Following the alignment stage, Qualimap 2 does a quality evaluation, and featureCounts or htseq-count performs feature counting [6]. The pipeline does a DE-analysis with DESeq2 after constructing a quantification matrix for the transcripts. DE-analysis generates three types of tables: normalised quantification tables, essential statistics for the transcript list, and a list of significantly differentially expressed transcripts (with an FDR of 0.05 as the default threshold). Salmon's Transcripts Per Million (TPM) scaled using the average transcript length across samples and then the library size by tximport was used to estimate the normalised values [7]. The DE-analysis results were visualised using a volcano plot (Log2 Fold Change vs p-value), plotted using R/Bioconductor package EnhancedVolcano [8]. The DE-lncRNAs across groups were also visualized using a heatmap (average normalised counts), generated by Morpheus version 1 [9].

**Network analysis and visualization**

The miRNAs interaction with the differentially expressed lncRNAs were established using miRNet 2.0 [10]. The interacting miRNAs were then used to find the genes interacting with the miRNAs, thus building an lncRNA-miRNA-mRNA interaction network. miRTarBase was used to find the genes interacting with miRNAs [11]. A betweenness filter of 0.05 and Minimum Network were used to simplify the dense network.

**Differential Gene Expression Analysis**

The trimmed reads were mapped with human genome reference GRCh38.p13 using Salmon as mentioned before. Differential gene expression analysis was performed using DESeq2, and genes that were significantly differentially expressed as well as present in the lncRNA-miRNA-mRNA interaction network were selected for further downstream analysis

**Gene Set Enrichment Analysis**

The differentially expressed genes interacting directly or indirectly with the lncRNAs were used to perform gene set enrichment using fgsea R package against Molecular signature database [12–14]. The pathways were plotted using the ggplot2 R package, against the p value, combined score and number of genes involved in the pathways.

**Repeat Elements Distribution**

The differentially expressed lncRNA as well as total lncRNA sequences were fetched from LNCipedia and used to find the repeat elements using rmblast in RepeatMasker web server [15]. Short Interspersed Nuclear Element (SINE) and Long Interspersed Nuclear Element (LINE) category of repeat elements were selected and plotted using ggridges packages in R 4.0.2 for visualisation.

**TFBS Prediction**

The lncRNA sequences were uploaded into the Ciiider tool for the prediction of transcription factor binding sites (TFBS) within sequences [16]. JASPAR, an online open-sourced manually curated database with transcription factors (TF) binding profiles as position frequency matrices (PFMs) was used to scan the sequences to predict TFBS. The deficit cut-off of 0.15 was used. Predicted TFBS within the genomic coordinates of repeat elements, LINEs and SINEs in the lncRNA sequences were filtered.

**Statistical Analysis**

Descriptive statistics are presented as median [interquartile range; IQR] and *n* (%) for continuous and categorical variables, respectively. The Mann–Whitney U test and Kruskal–Wallis test with Dunn’s correction for multiple comparisons were used to compare differences in continuous data between groups as appropriate. The Chi-square test was used for categorical variables. Statistical analyses were performed using Graphpad Prism version 9.2.0. Pearson correlation analysis was performed for the differentially expressed lncRNAs across mild,
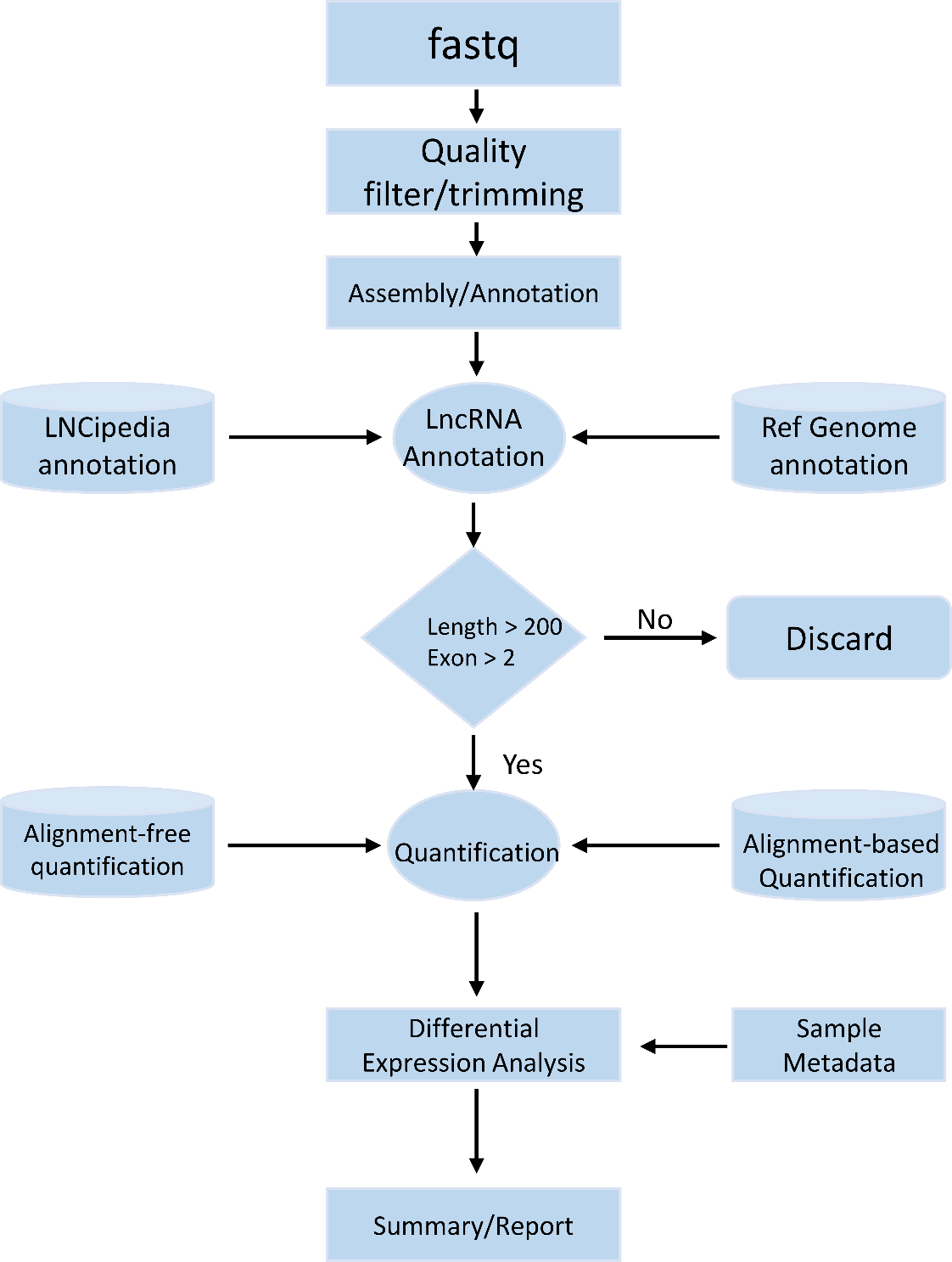
moderate, severe and mortality groups, using Morpheus version 1 [9].

**Supplementary Figure S1: Analytical workflow of the Identification and Differential Expression Analysis of lncRNA transcripts**. Each block represents one of the steps in our analysis pipeline. It makes use of lncRNA annotation data from LNCipedia ( <https://lncipedia.org/>) that has been predefined and highly curated, as well as human genome annotation data from GENCODE (https://www.gencodegenes.org/). To obtain a consensus and reliable lncRNA transcript for DEA analysis, all data is combined. It's worth noting that the pipeline uses custom scripts to filter intermediate findings and graphs and summaries the results using R scripts.

**Data Information Supporting Result Interpretation**

**Patient Cohort Characterization: Classification and Clinical Evaluation**

We recruited 117 hospitalized COVID-19 patients to understand the role of lncRNAs as a modulator of host response. The patients were stratified into specific groups based on their disease severity, outcomes and distinct disease phenotypes. Initially, all patients were categorized based on their outcomes into two groups: Recovered (n=71) and Mortality (n=15). The recovered patients were further stratified into three groups: Mild (n=46), Moderate (n=10) and Severe (n=15). Few samples with distinct clinical presentation were not falling under either of the subcategories, were stratified into two respiratory symptom based groups: respiratory support, RS (n=17) and shortness of breath, SOB (n=14). Briefly, we sequenced the transcriptome of 117 nasopharyngeal samples (~2.15 billion total reads, ~15.26 million mean reads/sample), followed by analysis for differential expression, lncRNA-miRNA-mRNA interaction, pathway enrichment, and role of regulatory sites with the repeat elements.

The demographics and clinical data of the 117 patients are summarized in **Supplementary Table S1,** wherein, specific clinical factors significantly correlate with difference in the disease severity and outcome. The median Ct value of *E* gene was significantly different (p value = 0.0255) between recovered (25.7) and mortality (20.53) patients. Similarly, the median Ct value of the RdRp gene was significantly different (p value = 0.029) between the RS (28.57) and the SOB (23.59) patients. The SpO_2_ level was significantly different between recovered/mortality and mild/moderate/severe (p value <0.0001 in both cases). The median age of the mild patients (49.5 years) was significantly lower (p value = 0.0228) than that of moderate, severe and mortality (~61 years). The number of patients requiring respiratory support were significantly different across recovered/mortality, mild/moderate/severe and RS/SOB (p value <0.0001 in all cases). The duration of hospital stay was significantly different in mild/moderate/severe and RS/SOB groups (p value = 0.0124 and 0.0114, respectively). Amongst the symptoms, sore throat and shortness of breath were significantly different in mild/moderate/severe and RS/SOB groups respectively (p value = <0.0001 and 0.0012, respectively).

**Supplementary Table S1: Demographic and Clinical characteristics of COVID-19 patients at the time of Hospital Admission**

|  | **Outcomes** | | | **Severity Sub-phenotypes** | | | | **Phenotype** | | |
| --- | --- | --- | --- | --- | --- | --- | --- | --- | --- | --- |
|  | Recovered (n=71) | Mortality (n=15) | **p-value** | Mild (n=46) | Moderate (n=10) | Severe (n=15) | **p-value** | SOB (n=14) | RS (n=17) | **p-value** |
| Age (years) | 57(30-66) | 61.5(53-68) | 0.1081^a^ | 49.5(16-78) | 60.5(44-84) | 61(36-80) | **0.0228^a^** | 45.5(18-74) | 58(23-84) | 0.0539^a^ |
| Male (%) | 46(64.78) | 9(64.28) |  | 56.5 | 90 | 73.3 |  | 64.3 | 52.9 |  |
| Female (%) | 25(35.21) | 5(35.71) |  | 43.5 | 10 | 26.7 |  | 35.7 | 47.1 |  |
| Ct value (E) | 25.7(21.63-28.9) | 20.535(18.43-26.05) | **0.0255^a^** | 25.41 | 26.9 | 25.7 | 0.8048^a^ | 23.67 | 27.31 | 0.0901^a^ |
| Ct value (RdRp) | 27.15(21.94-29.31) | 22.295(18.24-28) | 0.1020^a^ | 25.95 | 28.78 | 27.15 | 0.3339^a^ | 23.59 | 28.57 | **0.029^a^** |
| SpO_2_ | 97(91-98) | 89.5(85-94) | **0.0354^a^** | 97.5 | 91.5 | 80 | **< 0.0001^a^** | 97 | 96 | 0.4773^a^ |
| Respiratory support (%) | 22/71 | 15/15 | **<0.00001^c^** | 0/46 | 8/10 | 15/15 | **< 0.0001^a^** | 0/14 | 17/17 | **< 0.0001^b^** |
| Hospital stay (days) | 9(5-13) | 12(7-21) | 0.2521^a^ | 7 | 14 | 11 | **0.0124^a^** | 5.5 | 12 | **0.0114^a^** |
| Sore throat (%) | 26.8 | 0 | **0.034^c^** | 12 | 3 | 4 | 0.6426^b^ | 4 | 3 | 0.4691^d^ |
| Cough (%) | 47.9 | 40 | 0.5778^d^ | 22 | 4 | 8 | 0.8075^b^ | 7 | 7 | 0.6232^d^ |
| Fever (%) | 76.1 | 66.7 | 0 .4488^d^ | 34 | 7 | 13 | 0.5367^b^ | 11 | 10 | 0.2418^d^ |
| Body ache (%) | 12.6 | 26.7 | 0.1693^d^ | 8 | 1 | 0 | 0.2054^b^ | 2 | 3 | 0.8001^d^ |
| Shortness of breath (%) | 30.9 | 46.7 | 0.2431^d^ | 0 | 4 | 12 | **<0.0001^b^** | 14 | 8 | **0.0012^d^** |
| Other symptoms (%) | 33.8 | 33.3 | 0.9721^d^ | 17 | 4 | 3 | 0.4375^b^ | 4 | 5 | 0.9591^d^ |
| Thyroid disorder (%) | 15.5 | 13.3 | 0.8319^d^ | 5 | 1 | 5 | 0.1063^b^ | 1 | 2 | 0.6649^d^ |
| Asthma/COPD (%) | 8.5 | 0.0 | 0.5846^c^ | 2 | 2 | 2 | 0.2145^b^ | 0 | 1 | 0.3563^d^ |
| Diabetes (%) | 35.2 | 40.0 | 0. 7256^d^ | 13 | 3 | 9 | 0.0859^b^ | 4 | 5 | 0.9591^d^ |
| Hypertension (%) | 39.4 | 66.7 | 0.0536^d^ | 17 | 5 | 6 | 0.7752^b^ | 5 | 8 | 0.5241^d^ |
| Heart disease (%) | 14.1 | 20.0 | 0.5611^d^ | 8 | 0 | 2 | 0.3566^b^ | 3 | 3 | 0.7908^d^ |
| Kidney disorder (%) | 7.0 | 6.7 | 0.8730^d^ | 2 | 1 | 2 | 0.4606^b^ | 3 | 4 | 0.8893^d^ |
| Other comorbidities (%) | 15.5 | 13.3 | 0.8319^d^ | 8 | 1 | 2 | 0.7964^b^ | 2 | 3 | 0.8001^d^ |

*Patients’ segregation based on disease outcome and severity classification.*

*Data are shown as median (IQR) or n(%);* *^a^Mann Whitney U test;* *^b^Kruskal Wallis test;* ^c^*Fisher Exact test*; *^d^Chi2 test;* *Values of significance are highlighted in bold.*

**Heightened Inflammatory Response is Linked to COVID-19 Mortality Cases**

To understand the plausible role of lncRNA in regulating host response in the patients who succumbed to COVID-19, we analysed the lncRNA expression profile difference between the recovered and mortality groups. We found three lncRNAs significantly downregulated in the mortality patients (p-adjusted/FDR/q value ≤ 0.05, Log2 Fold Change ≥ ± 2) (**Additional File 2**). To look for other lncRNAs differentially expressed based on Log2 fold change and p value (although may not be significant based on q value), we performed hierarchical clustering of the differentially expressed (DE) lncRNAs (top 20 Log2 fold change, irrespective of p-adjusted value). We found LINC00174:11 to be 22.5 fold downregulated in the mortality group. LINC00174 is known to downregulate miR-1910-3p by acting as a competing endogenous RNA (ceRNA), and miR-1910-3p activates NF-kB signalling pathway [17, 18]. The downregulation of LINC00174 in the mortality group compared to the recovered possibly indicates an elevated NF-kB signalling pathway, which can lead to cytokine storm in the mortality patients.

ROR1-AS1:6 was downregulated in the mortality patients compared to the recovered ones. Studies have reported ROR1-AS1 to sponge miR-375 [19]. Downregulation of miR-375 is known to downregulate inflammatory cytokines such as interleukin-6 (IL-6), tumor necrosis factor-α (TNF-α), interleukin-1β (IL-1β) in macrophages [20]. Downregulation of miR-375 also causes downregulation of T-helper (Th) cell mediated immune response [21]. Besides, miR-375 is reported to be positively regulating viral replication [22–24]. Thus, downregulation of ROR1-AS1: in the mortality group indicate a possible heightened immune and inflammatory response as well as higher viral replication, evident by upregulation of TNF (p value = 0.003) and the lower Ct value compared to the recovered patients.

Another antisense RNA, RNASEH1-AS1:23, was downregulated in the mortality patients. RNASEH1-AS1 is reported to downregulate the expression of miR-218-5p [25]. A study reported miR-218-5p mimic downregulated CCL20 and IL-8 expression in NBHE cell lines [26]. miR-218-5p also downregulates inflammatory cytokines and NK cell mediated cytotoxicity which suppress the killing effect of NK cells [27]. Combined together, these indicate towards downregulation of chemokine ligand 20 (CCL20), IL-8 and other inflammatory cytokines. However, another study by Zhou et al reported that miR-218-5p positively regulates TNF-α, IL-1β, and monocyte chemotactic protein 1 (MCP-1), indicative of RNASEH1-AS1 downregulation mediated heightened inflammatory response in the mortality patients, which correlates with previous findings [28–31]. Interestingly, we also found several cytokine and interleukins (CCL3, CCL20, IL36A, IL1A) to be upregulated in the mortality group (**Additional file 5**). The same group also established the role of miR-218-5p in inducible nitric oxide synthase (iNOS) expression and cell death, which highlights the RNASEH1-AS1 downregulation mediated stress and apoptosis in the mortality patients.

We then looked into the lncRNA-miRNA-mRNA interaction to understand the possible biological functions of the differentially expressed lncRNAs, using miRNet 2.0 and miRTarBase [10, 11]. We found *CDKN1A* (downregulated in mortality, p value = 0.018), a virus infection-associated gene to interact with RNASEH1-AS1 through miR-345-5p/miR-576-5p/miR-942-5p. We also found *ACTB*, a housekeeping gene (downregulated in the mortality, p value = 0.015) to interact with RNASEH1-AS1 through miR-218-5p/miR-324-5p. While CDKN1A is involved in complement activation system, an important immune response mechanism, ACTB is involved in maintaining cell motility, structure, integrity and intracellular signalling [32]. To learn more about the biological significance of the lncRNA-gene interaction network, we performed gene set enrichment analysis on the differentially expressed genes against KEGG, and selected the pathways with significant differentially expressed genes. Inflammatory response, Interferon γ response, TGF-β signalling, TNF-α signalling and apoptosis related pathways were found to be enriched in the mortality group, indicating overall heightened inflammatory response and apoptosis in the mortality group.

**LncRNA Mediated Dysregulation of Immune Response in COVID-19 Sub-phenotypes**

To assess the role of lncRNAs in modulating host response to COVID-19 leading to different disease trajectories, we analysed for the differential expression of lncRNAs amongst COVID-19 sub-phenotypes. We investigated differential expression between mild vs moderate/severe/mortality, moderate vs severe/mortality and severe vs mortality patients. We found six significantly differentially expressed lncRNAs in mild vs moderate (one upregulated, five downregulated), four downregulated in mild vs severe, 13 (seven upregulated and six downregulated) in mild vs mortality, 15 (one upregulated and 14 downregulated) in moderate vs severe and 21 (four upregulated and 17 downregulated) in the severe vs mortality (**Additional File 2**).

We found LINC00294:1 to be highly upregulated (Log2FC: 26.47, q value 1.29E-08) in the moderate patients compared to the mild. The LINC00294 is reported to sponge the miR-1278, which inhibits the inflammatory cytokines IL-22 and CXCL14 [33, 34]. Another downregulated LINC00504:9 (Log2FC: -22.22, q value 1.57E-05) in the moderate patients is known to inhibit miR-140-5p, upregulation of which inhibits the inflammatory cytokines through MyD88/NF-kB pathway [35, 36]. Thus, the downregulation of LINC00504 indicates a possible decrease in inflammatory response in the moderate patients. Interestingly, CXCL14 was downregulated in our moderate group, compared to mild (p value = 0.01). IL6, an inflammatory interleukin was also downregulated in the moderate group (p value = 0.03). The downregulation of RNASEH1-AS1:23 in the moderate patients (Log2FC: -21.74, q value 2.67E-05) also indicates a decreased inflammatory response in the moderate. MALAT1, a highly conserved lncRNA across mammals, was also found to be downregulated (Log2FC: -22.79, q value 8.48E-06) in the moderate. MALAT1 is known to suppress the immune response through Maf and IL-10 expression in Th cells during infection [37]. Viral infection-induced downregulation of MALAT1 triggers IRF3 mediated type I IFN production and innate antiviral immune response [38]. These two pieces of evidence, along with downregulation of CXCL14 and IL6 suggest a lncRNA-mediated heightened immune response in the moderate compared to the milder patients. Thus, it seems to be a case of a fine balance between the heightened immune response and decreased inflammatory response which controls the severity in the moderate.

In the mild vs severe, UGDH-AS1:11 was found to be downregulated (Log2FC: -22.28, q value 1.34E-06) in the severe. The downregulation of UGDH-AS1 is correlated with upregulation of *EDN1*, a potent vasoconstrictor and involved in COVID-19 disease severity [39, 40]. We also observed EDN1 upregulation in the severe group (p value = 0.019). Besides, UGDH-AS1 also interacts with MOV10 and UPF1, two RNA helicase involved in antiviral immune response through IFN induction and NMD pathway [41–43]. Thus, it seems the downregulated UGDH-AS1 results in a decreased antiviral response and increased disease severity in the severe patients compared to the milder ones. We also found LINC00504:9 to be downregulated in the severe (Log2FC: - 23.81, q value 7.81E-08). Although the downregulation of LINC00504 indicates a decreased immune response, the interaction of LINC00504 with MOV10 suggests a decreased antiviral response in the severe patients [40].

In the mild vs mortality patients, we observed downregulation of MALAT1:9, LINC00504:9 and RNASEH1-AS1:23 in the mortality. The downregulation of LINC00504:9 and RNASEH1-AS1:23 indicate a decreased inflammatory and antiviral response in the mortality patients, whereas the downregulation of the MALAT1:9 indicates an increased innate immune response in the mortality, contrary to other findings [44–47]. LUCAT1:3, a negative feedback regulator of IFN response, was upregulated in our mortality patients (Log2FC: 6.01, q value 0.013), which indicates activation of interferon immunity [48, 49]. LINC01537:2, downregulated in the mortality, is known to upregulate Phosphodiesterase 2A (PDE2A), which is a negative regulator of iNOS and augments T cell activation [50–52]. Thus, the downregulation of LINC01537 reflects increased iNOS mediated stress and decreased T cell activation in the mortality compared to the mild patients.

Between moderate vs severe, we found MALAT1:9 to be upregulated in the severe patients (Log2FC: 21.06, q value 4.91E-09), indicating a decreased immune response in the severe. Similar to the mild vs severe sub-phenotypes, UGDH-AS1:11 was downregulated in the severe compared to the moderate patients, indicating a decreased antiviral response and increased disease severity in the severe. ROR1-AS1:6, an antisense transcript, was also found to be downregulated in the severe (Log2FC: -23.29, q value 1.84E-11), and is known to sponge miR-375, upregulation of which inhibits IFN-𝛾 induced program death 1 ligand 1 (PD-L1). Upregulation of PD-L1 facilitates cancer cells to escape the immune system. Moreover, miR-375 is reported to positively regulate the inflammatory cytokines such as TNF-ɑ and IL-6 [53–56]. Thus, the downregulation of ROR1-AS1 reflects possible miR-375 mediated inhibition of PD-L1 and increased level of inflammatory cytokines in the severe patients. The upregulation of inflammatory cytokines such as CXCL5, CXCL14 in the severe group (p value 0.049 and 0.010 respectively) is also indicative of the lncRNA-mediated increased inflammatory response in the severe group.

In the severe vs mortality patients, we found three subtypes of LINC00273, all were downregulated in the mortality. LINC00273 is reported to sponge miR-200a-3p, upregulation of which suppresses early innate immune response [57, 58]. Thus, the LINC00273 downregulation could possibly explain the decreased early innate immune response in the mortality patients.

**Correlated Expression of lncRNAs Between the COVID-19 Sub-phenotypes**

We then performed Pearson correlation analysis on the normalised counts of lncRNAs across sub-phenotypes to identify the lncRNA expression pattern. We found two clusters of lncRNAs in the mild vs mortality patients. We also obtained a cluster of nine lncRNAs, all of which were downregulated in the moderate vs severe. Another cluster of 10 lncRNAs, out of which six were overlapping with the moderate vs severe was found, where all 10 lncRNAs (TRPM2-AS:7, lnc-HLA-B-2:13, lnc-CMSS1-1:4, lnc-PDZD7-3:10, TMEM9B-AS1:1, lnc-ZNF708-2:12, LINC01537:2, lnc-FAM153C-5:15, ROR1-AS1:6, LINC00174:11) were downregulated in patients with higher degree of disease severity. This suggests a possible role of these lncRNAs in regulating disease severity. In another cluster of three lncRNAs (lnc-FRMD5-3:2, lnc-PALLD-3:1, lnc-MNX1-5:1), all were upregulated in the mortality compared to the severe. Lastly, a large cluster of 16 lncRNAs (lnc-STIM2-7:1, lnc-MSH3-2:1, lnc-KCNE1B-3:5, lnc-IRS4-2:1, lnc-PRR32-4:2, lnc-MYO16-7:1, lnc-UTY-17:1, lnc-KCNE1B-155:1, lnc-KCNE1B-3:3, LINC00273:1, lnc-GPR39-10:2, lnc-TMEM132C-11:1, lnc-KCNE1B-3:2, lnc-KCNE1B-3:4, LINC00273:11, LINC00273:13) was obtained, all of which were downregulated in the mortality patients, when compared to the severe, suggesting a possible association of these lncRNAs (from both the clusters) with COVID-19 mortality.

**LncRNA Mediating Respiratory Distress amongst COVID-19 Patients**

We then looked at the differential expression of lncRNAs between the SOB and RS patients, to understand the possible underlying lncRNA signatures which may lead to the requirement of respiratory support in a subset of patients despite having similar SpO_2_ level. We found one autophagy related antisense transcript RTCA-AS1:8 to be downregulated in the RS group compared to the SOB patients (**Additional File 2**). Although the biological role of the RTCA-AS1 is not well studied, a study reported decreased expression of RTCA-AS1 in the high-risk oropharyngeal squamous cell carcinoma patients [59]. Importantly, downregulation of the RTCA-AS1 in SARS-CoV-2 infected human bronchial organoid compared to the uninfected group has been reported [60]. We also compared the expression of the lncRNAs across mild, moderate, RS and SOB to understand how different they are, as based on the clinical parameters of the SOB and RS patients, they fall in between the mild and moderate. We found that the SOB patients were close to the mild while the RS were similar to the moderate in terms of the lncRNA expression. Observations seem to justify the segregation of the RS and SOB patients under a different functional analysis.

**lncRNA-miRNA-mRNA Interactome Across COVID-19 Sub-phenotypes**

We then constructed a lncRNA-miRNA-mRNA interaction network to understand the possible biological functions of the differentially expressed lncRNAs (**Supplementary** **Figure S2A-2E**). We identified few more lncRNAs from the interaction network, which, however, were not significantly differentially expressed (based on p-value) in the comparison groups. For example, we identified NEAT1 in the mild vs mortality patients, which we did not find through differential expression analysis. Based on differential gene expression analysis, we found CALM3, VAV3, WIPI2 and MAD2L1 to interact with LINC00294, a lncRNA differentially expressed in mild vs moderate comparison group (**Additional file 4**). Upregulation of LIC00294, CALM3, VAV3, WIPI2 and MAD2L1 in the moderate (vs mild) suggest a positive regulation of these genes by LINC00294 (**Supplementary Figure S2F-I**). CALM3 is reported to be involved in adaptive immune response in HIV, HSV, CMV infection [61]. VAV3 is reported to be upregulated in cystic fibrosis airway epithelial cells [62]. WIPI2 is known to be involved in autophagosome formation and pathogen clearance [63]. MAD2L1 is a component of mitotic spindle assembly, therefore, upregulation of MAD2L1 causes chromosome instability. Besides, we also found HSPA8 (upregulated in moderate, p value = 0.028) to interact with LINC00294 through miR-205-5p. HSPA8 is a HSP70 family member and is involved in viral protein folding and assembly [64]. IGF1R, upregulated in moderate and severe (p value = 0.011, 0.002 respectively), interacts with LINC00294 through miR-143-5p. IGF1R is known to be involved in IGF1 pathway which positively regulates inflammatory response during viral infection [65]. CD47, downregulated in moderate group (p value = 0.010), interacts with LINC00294 through miR-21-5p. CD47 is known to act as a checkpoint for immune response, therefore downregulation of CD47 suggests an immune-evasion by the pathogen [66]. The downregulation of CDKN1A in the mortality group (compared to recovered and mild, p value = 0.018, 0.04 respectively) and its interaction with RNASEH1-AS1 through miR-181a-5p indicates a decreased immune response in the mortality group. Finally, to understand the overall perturbation of biological pathways by the differentially expressed lncRNAs, we performed gene set enrichment analysis for the interacting genes (using expression rank from differential gene expression analysis) (**Supplementary Figure S2J-2M**). Interestingly, several immune and inflammatory pathways, such as interferon α/γ response, TNF-α signalling, IL6/JAK/STAT signalling, IL2/STAT3 signalling, PI3K/AKT signalling, inflammatory response, were significantly associated in all the comparison groups (except severe vs mortality). Besides, stress and apoptosis related pathways, such as p53 pathway, reactive oxygen species pathway, unfolded protein response pathway, were also enriched in mild vs moderate/mild vs severe and mild vs mortality comparison groups. The positive NES of inflammatory response, TNFα signalling, IL2/STST4 signalling, PI3K AKT signalling, TGFβ signalling, and interferon response, all of which are involved in inflammatory response, shows an overall dysregulation of inflammatory response in severe and mortality patients. Overall, the gene set enrichment analysis revealed a possible lncRNA-mediated dysregulated stress and immune response associated with COVID-19 disease severity.

**
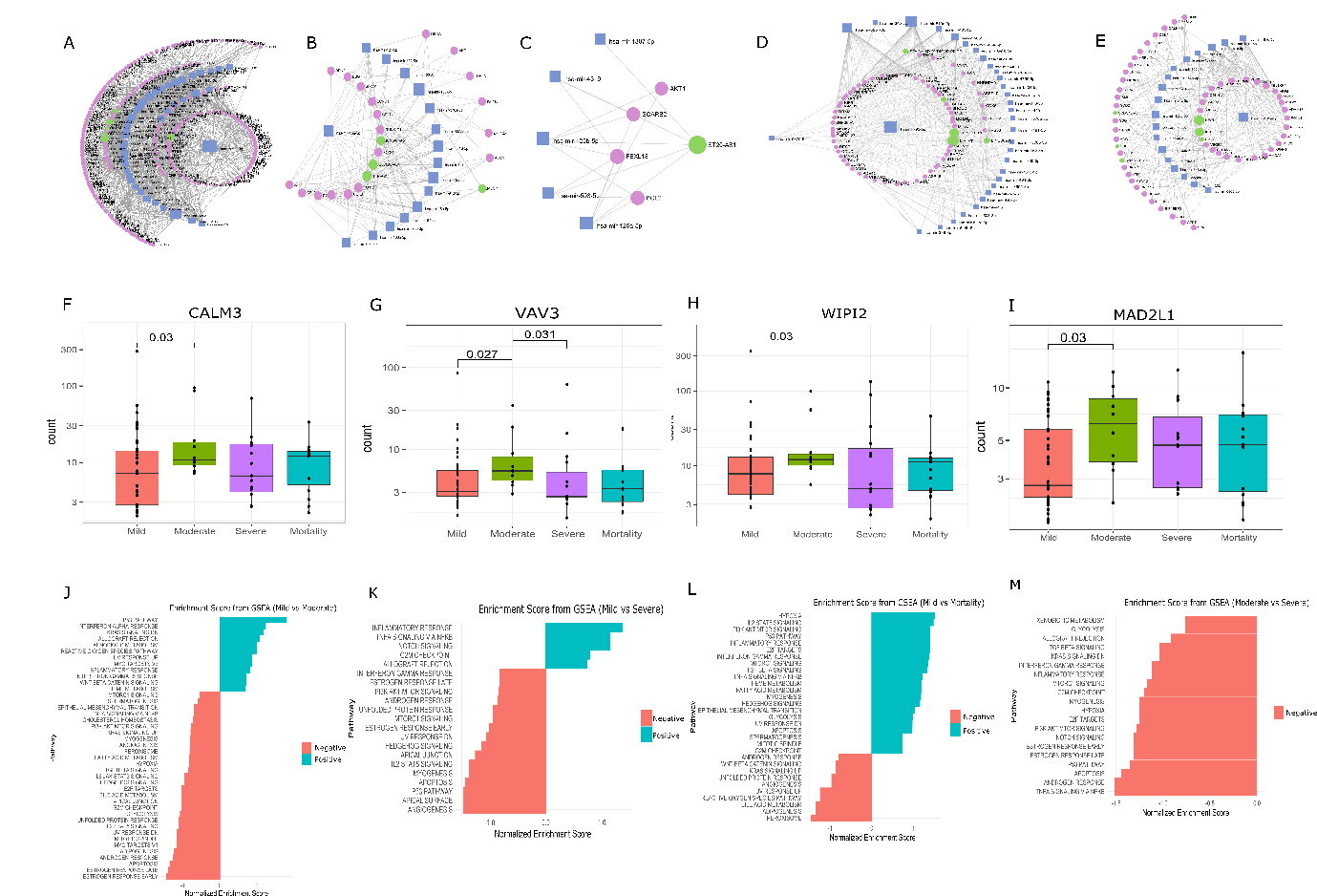
**

**Supplementary Figure S2: Functional analysis of the lncRNAs differentially expressed across COVID-19 sub-phenotype comparison groups.**

(A-E) lncRNA-miRNA-mRNA interaction of differentially expressed lncRNAs, where blue boxes represent miRNA, pink circles mRNAs and green circles lncRNAs, for (A) mild vs moderate, (B) mild vs severe, (C) severe vs mortality, (D) moderate vs severe, and (E) mild vs mortality. (F-I) Normalized expression of differentially expressed genes (F) CALM3, (G) VAV3, (H) H, and (I) MAD2L1, captured through lncRNA-miRNA-mRNA interactome. (J-M) Gene set enrichment analysis of the genes interacting with the differentially expressed lncRNAs, where x-axis represents the normalized enrichment score. (J) mild vs moderate, (K) mild vs severe, (L) mild vs mortality, (M) moderate vs severe patients. The colour highlights the direction of normalized enrichment score.

**Are Regulatory Motifs Within Repeat Elements Modulating the Identified lncRNAs?**

To understand the possible mechanism of gene regulation by the specific lncRNA, we looked into the repeat element distribution within the differentially expressed lncRNAs. We used rmblast in the RepeatMasker web server to find the repeat elements within the lncRNAs [15]. Based on the relative abundance (with respect to total repeat elements within differentially expressed lncRNA) (**Supplementary Figure S3A**) and available literature, we then selected the most studied, Short Interspersed Nuclear Elements (SINE) and Long Interspersed Nuclear Elements (LINE) repeat elements present in the differentially expressed lncRNAs. We found six sub-types of LINEs (LINE/L1, LINE/L2, LINE/CR1, LINE/RTE-X) and two sub-type of SINEs (SINE/Alu, SINE/MIR) across all the lncRNAs. Density plots of the repeat element distribution in the lncRNAs (normalised for the size of lncRNA) across groups’ shows a statistically significant higher distribution of SINE/Alu (p value < 0.0001), SINE/MIR (p value 0.0014) and LINE/L1 (p value < 0.0001) elements across the comparison groups. We also plotted the frequency distribution of the repeat elements within the lncRNAs. It is important to note that a higher presence of Alu elements, within the SINEs, was found in the differentially expressed lncRNAs in the mortality patients, conforming to the increased stress response in the mortality group in our data and existing literature suggesting the role of Alu repeats in stress response [67–69]. Interestingly, we observed a higher abundance (in terms of total length of lncRNA) SINE and LINE within the differentially expressed lncRNA when compared to the overall abundance of the repeat elements in LNCipedia database (**Supplementary Figure S3B**). This indicate that the higher abundance of the LINE and SINE is not due to their high abundance within lncRNA, rather it is indeed associated with COVID-19 disease severity.

We then predicted the transcription factor binding sites (TFBS) within the repeat elements present within the lncRNAs, to explore the possible mechanism of the repeat element-mediated gene regulatory function (upstream and downstream genes) of the lncRNAs. We filtered the total TFBS to identify TFBS within the repeat element region of the unique lncRNAs (with known biological functions) differentially expressed across all sub-phenotypes. Subsequently, we narrowed down to seven lncRNAs (LINC00294:1, LINC00504:9, RNASEH1-AS1:23, TRPM2-AS:7, UGDH-AS1:11, ROR1-AS1:6, and LINC00174:11) from a total of 12 lncRNAs. We then looked into the genes present within 5 kb upstream and downstream of the seven lncRNAs (12 genes), and performed a pathway enrichment analysis to understand the biological functions of the genes. Interestingly, we found two pathways with direct association with infectious disease, NOD-like receptor signalling pathway and coronavirus disease pathway. While *TRPM2* gene (present within ±5kb of TRPM2-AS1:7) was involved in the NOD-like receptor signalling pathway, *RPS7* (present within ±5kb of RNASEH1-AS1:23) gene was involved in the coronavirus disease pathway.


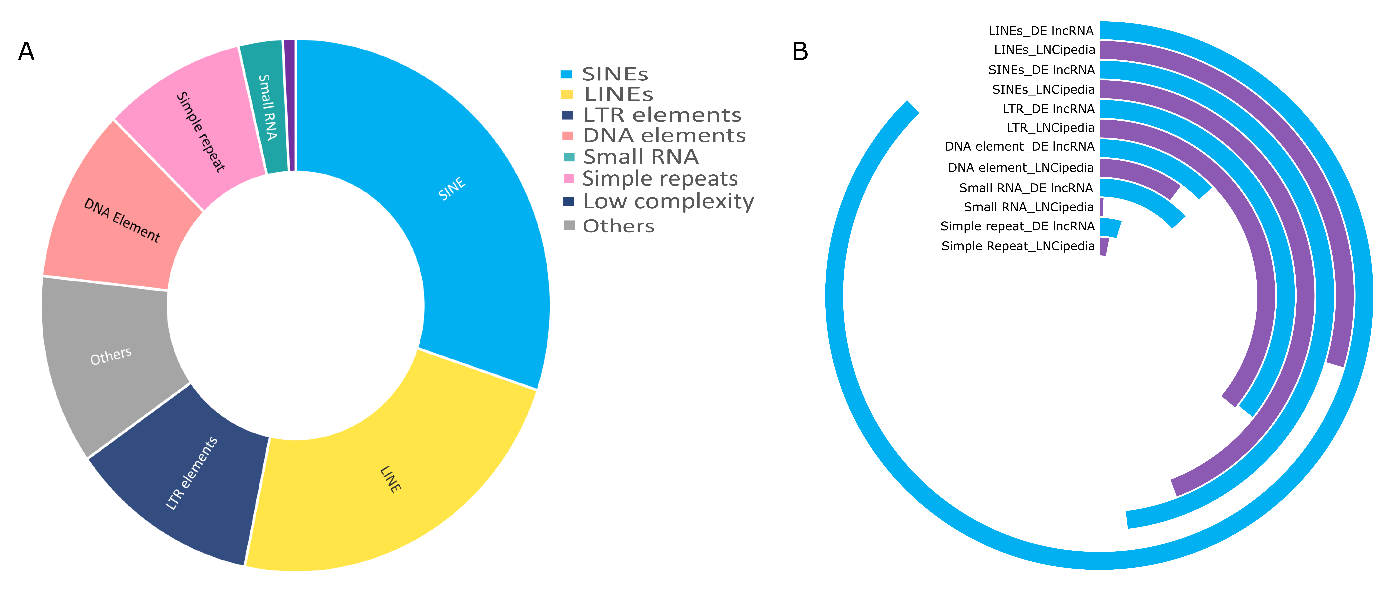


**Supplementary Figure S3: Repeat element distribution across all category**

(A) Repeat element abundance (% of total repeat element) of each class. (b) Repeat element distribution (% of total lncRNA sequence) across differentially expressed lncRNAs and LNCipedia database

Finally, we looked at the TFBS within the repeat elements of these two lncRNAs for possible transcription factor mediated regulation of the overlapping genes *TRPM2* and *RPS7*. We found SOX2, a transcription factor that binds to the Alu and L1 elements within TRPM2-AS1, positively correlates with TRPM2 expression while GATA3 negatively regulates TRPM2 expression [70, 71]. Another study indicates a positive correlation of FOXO1 and FOXO3 with TRPM2 expression [72]. GATA6 and SOX10, two transcription factors that bind to the Alu and L1 elements within RNASEH1-AS1, positively correlate with RPS7 expression [73, 74]. Although these pieces of evidence are not from the context of infectious disease, but they suggest a possible mechanism of repeat element-mediated gene expression regulation by the lncRNAs identified in the study.

**Detailed Description of Inferences Pertaining to the Study**

The transcriptomic analysis of the COVID-19 sub-phenotypes at an early stage of infection revealed lncRNA mediated dysregulation in immune response cascade. Our study has been able to highlight the role of lncRNAs in modulating the immune and stress response, which broadly correlates with COVID-19 disease severity. Although a limited number of studies reported differential expression of lncRNAs in COVID-19 cases, they are mainly focused on the differential expression of lncRNAs in a healthy vs disease scenario, but don't provide insights about lncRNA-mediated modulation of disease sub-phenotypes: mild, moderate, severe and mortality [75–78]. In this study we tried to answer an important unexplored host response factor: does noncoding RNA/s modulate the COVID-19 disease severity sub-phenotypes?

Through our transcriptome-based analysis of the hospital admitted patient cohort, we found that the distinct lncRNA expression profile could be associated with the pathological presentation of COVID-19 patients. This was supplemented by network, pathway, association, repeat elements and regulatory sites presence analysis to understand the plausible mechanism of the observations.

In patients with SARS-CoV-2 infection, several lncRNAs play major regulatory roles in the process of virus infection. MALAT1 and NEAT1, for example, have been demonstrated to be highly related to immunological responses and may be implicated in the inflammatory course of SARS-CoV-2 infected cells [79, 80]. Studies reported that MALAT1 was upregulated in COVID-19 patients as compared to healthy whereas it was downregulated in mild COVID-19 BAL cells [77, 80]. The known function of MALAT1 is to suppress the immune response through Maf and IL-10 expression in Th cells during infection whereas its downregulation triggers IRF3 mediated type I IFN production and innate antiviral immune response upon viral infection [37, 38]. In our study, MALAT1 was found to be downregulated in the moderate and mortality group whereas it was upregulated in the severe group. This evidence together with the present results suggests that reduced expression of MALAT1 induces a heightened immune response in moderate and mortality COVID-19 patients whereas its upregulation indicates a decreased immune response in the severe group. Another lncRNA, UGDH-AS1 was found to be downregulated in the severe group. UGDH-AS1 is known to interact with MOV10 and UPF1, two RNA helicases involved in the antiviral immune response through IFN induction and the NMD pathway and its downregulation has been correlated with upregulated EDN1 expression, a potent vasoconstrictor and involved in COVID-19 disease severity [40, 41, 43]. This evidence suggests that in severe individuals, downregulation of UGDH-AS1 leads to reduced antiviral response and an increase in disease severity as compared to milder patients. Previously reported LUCAT1 was upregulated in monocytes of severe COVID-19 patients suggesting a systemic interferon immunity against COVID-19 infection due to its role in negative feedback regulation of interferon response in human myeloid cells [48, 49].

In addition to known lncRNAs such as MALAT1, NEAT1, UGDH-AS1, and LUCAT1, we found several novel lncRNAs, differentially expressed across sub-phenotypes of COVID-19 patients where their expression profiles could be associated with the pathological presentation of COVID-19 patients. LINC00174:11, LINC00504:9, LINC00273, ROR1-AS1:6, and RNASEH1-AS1:23 which were previously not known to be associated with COVID-19, were downregulated in the mortality group. The downregulation of LINC00504:9 in the moderate, severe and mortality group (vs mild) indicate a possible miR-140-5p mediated decreased immune response. Downregulation of LINC00174 in the mortality group indicate a possible miR-1910-3p-mediated elevated NF-kB signalling, which is involved in the cytokine storm. Multiple reports also suggest elevated cytokine storm in COVID-19 mortality group. Besides, ROR1-AS1 downregulation in the mortality and severe group also suggest a miR-375-mediated increase in cytokine response. The downregulation of LINC00273 in the mortality group also suggests a miR-200a-3p-mediated decreased immune response. The downregulation of RNASEH1-AS1 in mortality group also suggest decreased immune response. Thus, the downregulation of LINC00174:11, LINC00504:9, LINC00273, ROR1-AS1:6, and RNASEH1-AS1:23 in the severe and mortality group, along with the differentially expressed genes captured through differential expression analysis and lncRNA-miRNA-mRNA interactome suggest a heightened inflammatory and decreased immune response in the severe and mortality group.

The Pearson correlation analysis revealed multiple clusters of lncRNAs associated with disease severity, thus indicating the possible functions of the lncRNAs in modulating disease severity. For example, LINC01684:22, lnc-H2BFWT-18:1, lnc-RWDD3-5:21, TRPM2-AS:7, lnc-HLA-B-2:13, lnc-CMSS1-1:4, lnc-PDZD7-3:10, TMEM9B-AS1:1 and lnc-ZNF708-2:12 were downregulated in the severe patients compared to the moderate. The significant correlation of the expression of these lncRNAs suggests their role in increasing disease severity. Based on the lncRNA-miRNA-mRNA interaction network and differential gene expression analysis, we observed a broad dysregulation of immune response, apoptosis, and stress response in the COVID-19 disease severity sub-phenotypes. Besides, we also found several immune-response related genes to be differentially expressed (not captured through lncRNA-miRNA-mRNA interaction network) across comparison groups. For example, MUC21 and HSPA1A, both were downregulated in the moderate and severe group compared to mild. While MUC21 provides mucosal immunity to SARS-CoV-2 infection, HSPA1A acts as an anti-inflammatory heat shock protein of HSP70 family [81–83]. MUC21 was also downregulated in the mortality group compared to the recovered. We also identified some inflammatory cytokine, chemokine and interleukins to be differentially expressed across groups. For example, we found downregulation of CCL5, CXCL1, IL26 in the moderate group, highlighting the reduced inflammatory response in the moderate group. IL6 and CXCL1, both were downregulated in the severe group. Finally, we observed an elevated inflammatory cytokines such as CXCL22, CCL20, CCL3 in the mortality group compared to the mild. In summary, we observed an initial increase in the immune response and decreased inflammatory response in the moderate, and then a decrease in immune response and antiviral response with increase in disease severity.

We also explored the abundance of the repeat elements in the discovered lncRNAs to understand the mechanism/s of lncRNA-mediated gene expression regulation. Several studies reported the gene regulatory role of the repeat elements, especially the transposable elements (TEs), i.e., LINEs and SINEs [67, 84, 85]. Interestingly, we found higher distribution of Alu repeats to be associated with COVID-19 disease severity. To our knowledge, this is the first study reporting the association of Alu repeats with COVID-19 disease severity. We also found a higher distribution of SINE/MIR (tRNA derived SINE) and LINE/L1 to be associated with COVID-19 severity. We found SINEs/LINEs in seven unique lncRNAs (LINC00294:1, LINC00504:9, RNASEH1-AS1:23, TRPM2-AS:7, UGDH-AS1:11, ROR1-AS1:6, and LINC00174:11) differentially expressed across all comparisons. Interestingly, earlier studies suggested the association of the Alu and L1 repeats in multiple diseases, including macular degeneration, tuberculosis and HIV infection. Bouttier et al reported high distribution of primate specific Alu repeats (40%) in *M. tuberculosis* infected cells [86]. Another group reported an increase in L1 retrotransposons in HIV infected primary CD4+ cells, with possible implications in innate immune response to HIV infection [87]. Apart from infectious disease, Alu element is also reported to be involved in diseases like macular degeneration, where DICER mediated increase of Alu RNA induces cytotoxicity in retinal pigmented epithelium cells by activating NLRP3 inflammasome and triggering TLR-independent Myd88 signalling during geographic atrophy [88, 89]. To understand the possible implications of the distribution of LINEs and SINEs within these seven lncRNAs, we looked into the biological functions of the genes present within 5kb upstream and downstream of these lncRNAs. Importantly, we found Nod-like receptor signalling pathway and coronavirus disease pathway to be biologically significant in the context of infectious disease.

Often, the repeat element-based gene regulations are mediated by the TFBS present within the repeat elements. For instance, binding of LXR within Alu repeat regulates the cholesterol metabolism, the active source of carbon for *M. tuberculosis* [86]*.* Another study reported the binding of HSF in Alu repeat to regulate stress response [67]. Here we explored the TFBS within the repeat elements in the lncRNAs and its possible implications for the genes present within 5kb upstream and downstream of the lncRNAs. Based on the pathway enrichment analysis, we selected TRPM2-AS1 and RNASEH1-AS1 and explored the role of the transcription factors that bind on the TFBS within the repeat elements, on the overlapping genes. We found four transcription factors (majorly bind to Alu and L1 repeat elements within TRPM2-AS1), SOX2, GATA3, FOXO1 and FOXO3, to regulate the TRPM2 gene expression, whilst transcription factors, SOX10 and GATA6 binds with RNASEH1-AS1 to regulate the RPS7 expression. These findings highlight, in a limited way, how the repeat elements within lncRNAs might regulate the expression of the overlapping genes. Future studies would be helpful to further expand on this. This is particularly important to understand the role of the lncRNAs in modulating COVID-19 disease severity.

Besides the lncRNAs with known biological function, we found several lncRNAs (for example, lnc-VN1R2-5:7, LINC01537:2, TRPM2-AS:7, lnc-SETSIP-2:10, UFL1-AS1:3, lnc-LRR1-1:3, lnc-CCNB1IP1-1:5, lnc-HLA-B-2:13), which are significantly differentially expressed in multiple comparison groups. Although the Log2 fold change and the q value suggest a strong possibility of their role in modulating the disease severity, the biological function of these lncRNAs are yet to be explored and are potent candidates for functional elucidation. Also, our differential expression analysis reveals the transcript-resolved differentially expressed lncRNAs, however, the existing knowledge of the biological functions of the lncRNAs do not provide the transcript-specific biological functions of the lncRNAs. Thus, a detailed exploration of the role of the lncRNA at transcript-level granularity will greatly improve our current understanding of the role of lncRNAs in modulating disease severity.

Future studies in this direction would benefit with longitudinal sampling of the patients wherein we could track the expression level differences of the specific lncRNAs during the patients stay in the hospital. It would be important to understand this further as we are experiencing different waves of the COVID-19 driven by the different VOCs – Alpha, Delta, Omicron. Nonetheless, it provides an important resource for the future studies being conducted in our lab inclusive of the single cell genomics-based approach.

Bibliography

1. Mehta P, Sahni S, Siddiqui S, Mishra N, Sharma P, Sharma S, et al. Respiratory Co-Infections: Modulators of SARS-CoV-2 Patients’ Clinical Sub-Phenotype. Frontiers in Microbiology. 2021.

2. Love MI, Huber W, Anders S. Moderated estimation of fold change and' ' dispersion for RNA-seq data with DESeq2. Genome Biol. 2014;15:550. doi:10.1186/s13059-014-0550-8.

3. Bolger AM, Lohse M, Usadel B. Trimmomatic: a flexible trimmer for Illumina sequence data. Bioinformatics. 2014;30:2114–2120. doi:10.1093/bioinformatics/btu170.

4. Kim D, Paggi JM, Park C, Bennett C, Salzberg SL. Graph-based genome alignment and genotyping with HISAT2 and HISAT-genotype. Nat Biotechnol. 2019;37:907–915. doi:10.1038/s41587-019-0201-4.

5. Kim D, Langmead B, Salzberg SL. HISAT: a fast spliced aligner with low memory requirements. Nat Methods. 2015;12:357–360. doi:10.1038/nmeth.3317.

6. Okonechnikov K, Conesa A, García-Alcalde F. Qualimap 2: advanced multi-sample quality control for high-throughput sequencing data. Bioinformatics. 2016;32:292–294. doi:10.1093/bioinformatics/btv566.

7. Patro R, Duggal G, Love MI, Irizarry RA, Kingsford C. Salmon provides fast and bias-aware quantification of transcript expression. Nat Methods. 2017;14:417–419. doi:10.1038/nmeth.4197.

8. Blighe K, Rana S, Lewis M. EnhancedVolcano: Publication-ready volcano plots with enhanced colouring and labeling. R package version. 2019;1.

9. Morpheus. https://software.broadinstitute.org/morpheus/. Accessed 28 Jan 2022.

10. Chang L, Zhou G, Soufan O, Xia J. miRNet 2.0: network-based visual analytics for miRNA functional analysis and systems biology. Nucleic Acids Res. 2020;48:W244–W251. doi:10.1093/nar/gkaa467.

11. Huang H-Y, Lin Y-C-D, Li J, Huang K-Y, Shrestha S, Hong H-C, et al. miRTarBase 2020: updates to the experimentally validated microRNA-target interaction database. Nucleic Acids Res. 2020;48:D148–D154. doi:10.1093/nar/gkz896.

12. Korotkevich G, Sukhov V, Budin N, Shpak B, Artyomov MN, Sergushichev A. Fast gene set enrichment analysis. BioRxiv. 2016. doi:10.1101/060012.

13. Subramanian A, Tamayo P, Mootha VK, Mukherjee S, Ebert BL, Gillette MA, et al. Gene set enrichment analysis: a knowledge-based approach for interpreting genome-wide expression profiles. Proc Natl Acad Sci USA. 2005;102:15545–15550. doi:10.1073/pnas.0506580102.

14. Liberzon A, Birger C, Thorvaldsdóttir H, Ghandi M, Mesirov JP, Tamayo P. The Molecular Signatures Database (MSigDB) hallmark gene set collection. Cell Syst. 2015;1:417–425. doi:10.1016/j.cels.2015.12.004.

15. Tarailo-Graovac M, Chen N. Using RepeatMasker to identify repetitive elements in genomic sequences. Curr Protoc Bioinformatics. 2009;Chapter 4:Unit 4.10. doi:10.1002/0471250953.bi0410s25.

16. Gearing LJ, Cumming HE, Chapman R, Finkel AM, Woodhouse IB, Luu K, et al. CiiiDER: A tool for predicting and analysing transcription factor binding sites. PLoS One. 2019;14:e0215495. doi:10.1371/journal.pone.0215495.

17. Shen Y, Gao X, Tan W, Xu T. STAT1-mediated upregulation of lncRNA LINC00174 functions a ceRNA for miR-1910-3p to facilitate colorectal carcinoma progression through regulation of TAZ. Gene. 2018;666:64–71. doi:10.1016/j.gene.2018.05.001.

18. Wang B, Mao J-H, Wang B-Y, Wang L-X, Wen H-Y, Xu L-J, et al. Exosomal miR-1910-3p promotes proliferation, metastasis, and autophagy of breast cancer cells by targeting MTMR3 and activating the NF-κB signaling pathway. Cancer Lett. 2020;489:87–99. doi:10.1016/j.canlet.2020.05.038.

19. Wang FZ, Zhang MQ, Zhang L, Zhang MC. Long non-coding RNA ROR1-AS1 enhances colorectal cancer metastasis by targeting miR-375. Eur Rev Med Pharmacol Sci. 2019;23:6899–6905. doi:10.26355/eurrev_201908_18729.

20. Ke Q-H, Chen H-Y, He Z-L, Lv Z, Xu X-F, Qian Y-G, et al. Silencing of microRNA-375 affects immune function in mice with liver failure by upregulating astrocyte elevated gene-1 through reducing apoptosis of Kupffer cells. J Cell Biochem. 2019;120:253–263. doi:10.1002/jcb.27338.

21. Biton M, Levin A, Slyper M, Alkalay I, Horwitz E, Mor H, et al. Epithelial microRNAs regulate gut mucosal immunity via epithelium-T cell crosstalk. Nat Immunol. 2011;12:239–246. doi:10.1038/ni.1994.

22. Bierkens M, Krijgsman O, Wilting SM, Bosch L, Jaspers A, Meijer GA, et al. Focal aberrations indicate EYA2 and hsa-miR-375 as oncogene and tumor suppressor in cervical carcinogenesis. Genes Chromosomes Cancer. 2013;52:56–68. doi:10.1002/gcc.22006.

23. Wang X, Jia Y, Wang X, Wang C, Lv C, Li X, et al. MiR-375 Has Contrasting Effects on Newcastle Disease Virus Growth Depending on the Target Gene. Int J Biol Sci. 2019;15:44–57. doi:10.7150/ijbs.25106.

24. Liu Y, Wang Q, Wen J, Wu Y, Man C. MiR-375: A novel multifunctional regulator. Life Sci. 2021;275:119323. doi:10.1016/j.lfs.2021.119323.

25. Jia J, Wang J, Yu J, Gao P, Liu Y, Li Y, et al. The positive feedback loop of RNASEH1-AS1/has-miR-218-5p/NET1 mediated by POU2F1 contributes to the development and progression of human lung squamous carcinoma. 2020. doi:10.21203/rs.3.rs-18125/v1.

26. Conickx G, Mestdagh P, Avila Cobos F, Verhamme FM, Maes T, Vanaudenaerde BM, et al. MicroRNA Profiling Reveals a Role for MicroRNA-218-5p in the Pathogenesis of Chronic Obstructive Pulmonary Disease. Am J Respir Crit Care Med. 2017;195:43–56. doi:10.1164/rccm.201506-1182OC.

27. Yang Q, Li J, Hu Y, Tang X, Yu L, Dong L, et al. MiR-218-5p Suppresses the Killing Effect of Natural Killer Cell to Lung Adenocarcinoma by Targeting SHMT1. Yonsei Med J. 2019;60:500–508. doi:10.3349/ymj.2019.60.6.500.

28. Karki R, Sharma BR, Tuladhar S, Williams EP, Zalduondo L, Samir P, et al. Synergism of TNF-α and IFN-γ Triggers Inflammatory Cell Death, Tissue Damage, and Mortality in SARS-CoV-2 Infection and Cytokine Shock Syndromes. Cell. 2021;184:149–168.e17. doi:10.1016/j.cell.2020.11.025.

29. Moradian N, Gouravani M, Salehi MA, Heidari A, Shafeghat M, Hamblin MR, et al. Cytokine release syndrome: inhibition of pro-inflammatory cytokines as a solution for reducing COVID-19 mortality. Eur Cytokine Netw. 2020;31:81–93. doi:10.1684/ecn.2020.0451.

30. Bülow Anderberg S, Luther T, Berglund M, Larsson R, Rubertsson S, Lipcsey M, et al. Increased levels of plasma cytokines and correlations to organ failure and 30-day mortality in critically ill Covid-19 patients. Cytokine. 2021;138:155389. doi:10.1016/j.cyto.2020.155389.

31. Zhu H, Wang X, Chen S. Downregulation of MiR-218-5p Protects Against Oxygen-Glucose Deprivation/Reperfusion-Induced Injuries of PC12 Cells via Upregulating N-myc Downstream Regulated Gene 4 (NDRG4). Med Sci Monit. 2020;26:e920101. doi:10.12659/MSM.920101.

32. Hu K, Li J, Wu G, Zhou L, Wang X, Yan Y, et al. The novel roles of virus infection-associated gene CDKN1A in chemoresistance and immune infiltration of glioblastoma. Aging (Albany, NY). 2021;13:6662–6680. doi:10.18632/aging.202519.

33. Zhou X, Lv L, Zhang Z, Wei S, Zheng T. LINC00294 negatively modulates cell proliferation in glioma through a neurofilament medium-mediated pathway via interacting with miR-1278. J Gene Med. 2020;22:e3235. doi:10.1002/jgm.3235.

34. Liu D, Qiao C, Luo H. MicroRNA-1278 ameliorates the inflammation of cardiomyocytes during myocardial ischemia by targeting both IL-22 and CXCL14. Life Sci. 2021;269:118817. doi:10.1016/j.lfs.2020.118817.

35. Hou T, Ye L, Wu S. Knockdown of LINC00504 Inhibits the Proliferation and Invasion of Breast Cancer via the Downregulation of miR-140-5p. Onco Targets Ther. 2021;14:3991–4003. doi:10.2147/OTT.S294965.

36. Yang Y, Liu D, Xi Y, Li J, Liu B, Li J. Upregulation of miRNA-140-5p inhibits inflammatory cytokines in acute lung injury through the MyD88/NF-κB signaling pathway by targeting TLR4. Exp Ther Med. 2018;16:3913–3920. doi:10.3892/etm.2018.6692.

37. Hewitson JP, West KA, James KR, Rani GF, Dey N, Romano A, et al. Malat1 Suppresses Immunity to Infection through Promoting Expression of Maf and IL-10 in Th Cells. J Immunol. 2020;204:2949–2960. doi:10.4049/jimmunol.1900940.

38. Liu W, Wang Z, Liu L, Yang Z, Liu S, Ma Z, et al. LncRNA Malat1 inhibition of TDP43 cleavage suppresses IRF3-initiated antiviral innate immunity. Proc Natl Acad Sci USA. 2020;117:23695–23706. doi:10.1073/pnas.2003932117.

39. Gattinoni L, Coppola S, Cressoni M, Busana M, Rossi S, Chiumello D. COVID-19 Does Not Lead to a “Typical” Acute Respiratory Distress Syndrome. Am J Respir Crit Care Med. 2020;201:1299–1300. doi:10.1164/rccm.202003-0817LE.

40. Turjya RR, Khan MA-A-K, Mir Md Khademul Islam AB. Perversely expressed long noncoding RNAs can alter host response and viral proliferation in SARS-CoV-2 infection. Future Virol. 2020;15:577–593. doi:10.2217/fvl-2020-0188.

41. Cuevas RA, Ghosh A, Wallerath C, Hornung V, Coyne CB, Sarkar SN. MOV10 Provides Antiviral Activity against RNA Viruses by Enhancing RIG-I-MAVS-Independent IFN Induction. J Immunol. 2016;196:3877–3886. doi:10.4049/jimmunol.1501359.

42. Balistreri G, Bognanni C, Mühlemann O. Virus Escape and Manipulation of Cellular Nonsense-Mediated mRNA Decay. Viruses. 2017;9. doi:10.3390/v9010024.

43. Gregersen LH, Schueler M, Munschauer M, Mastrobuoni G, Chen W, Kempa S, et al. MOV10 Is a 5’ to 3' RNA helicase contributing to UPF1 mRNA target degradation by translocation along 3' UTRs. Mol Cell. 2014;54:573–585. doi:10.1016/j.molcel.2014.03.017.

44. Hojyo S, Uchida M, Tanaka K, Hasebe R, Tanaka Y, Murakami M, et al. How COVID-19 induces cytokine storm with high mortality. Inflamm Regen. 2020;40:37. doi:10.1186/s41232-020-00146-3.

45. Li D, Chen Y, Liu H, Jia Y, Li F, Wang W, et al. Immune dysfunction leads to mortality and organ injury in patients with COVID-19 in China: insights from ERS-COVID-19 study. Signal Transduct Target Ther. 2020;5:62. doi:10.1038/s41392-020-0163-5.

46. Forlano R, Mullish BH, Mukherjee SK, Nathwani R, Harlow C, Crook P, et al. In-hospital mortality is associated with inflammatory response in NAFLD patients admitted for COVID-19. PLoS One. 2020;15:e0240400. doi:10.1371/journal.pone.0240400.

47. Abers MS, Delmonte OM, Ricotta EE, Fintzi J, Fink DL, de Jesus AAA, et al. An immune-based biomarker signature is associated with mortality in COVID-19 patients. JCI Insight. 2021.

48. Agarwal S, Vierbuchen T, Ghosh S, Chan J, Jiang Z, Kandasamy RK, et al. The long non-coding RNA LUCAT1 is a negative feedback regulator of interferon responses in humans. Nat Commun. 2020;11:6348. doi:10.1038/s41467-020-20165-5.

49. Aznaourova M, Schmerer N, Janga H, Zhang Z, Pauck K, Hoppe J, et al. Single cell RNA-seq uncovers the nuclear decoy lincRNA PIRAT as a regulator of systemic monocyte immunity during COVID-19. BioRxiv. 2021. doi:10.1101/2021.11.05.467458.

50. Gong W, Yang L, Wang Y, Xian J, Qiu F, Liu L, et al. Analysis of Survival-Related lncRNA Landscape Identifies A Role for LINC01537 in Energy Metabolism and Lung Cancer Progression. Int J Mol Sci. 2019;20. doi:10.3390/ijms20153713.

51. Kurelic R, Krieg PF, Sonner JK, Bhaiyan G, Ramos GC, Frantz S, et al. Upregulation of Phosphodiesterase 2A Augments T Cell Activation by Changing cGMP/cAMP Cross-Talk. Front Pharmacol. 2021;12:748798. doi:10.3389/fphar.2021.748798.

52. Rentsendorj O, D’Alessio FR, Pearse DB. Phosphodiesterase 2A is a major negative regulator of iNOS expression in lipopolysaccharide-treated mouse alveolar macrophages. J Leukoc Biol. 2014;96:907–915. doi:10.1189/jlb.3A0314-152R.

53. Zhao S-P, Yu C, Xiang K-M, Yang M-S, Liu Z-L, Yang B-C. miR-375 Inhibits Autophagy and Further Promotes Inflammation and Apoptosis of Acinar Cells by Targeting ATG7. Pancreas. 2020;49:543–551. doi:10.1097/MPA.0000000000001536.

54. Lei L, Zhou C, Yang X, Li L. Down-regulation of microRNA-375 regulates adipokines and inhibits inflammatory cytokines by targeting AdipoR2 in non-alcoholic fatty liver disease. Clin Exp Pharmacol Physiol. 2018;45:819–831. doi:10.1111/1440-1681.12940.

55. Wu Q, Zhao Y, Sun Y, Yan X, Wang P. miR-375 inhibits IFN-γ-induced programmed death 1 ligand 1 surface expression in head and neck squamous cell carcinoma cells by blocking JAK2/STAT1 signaling. Oncol Rep. 2018;39:1461–1468. doi:10.3892/or.2018.6177.

56. Xu N, Qiao L, Yin L, Li H. Long noncoding RNA ROR1-AS1 enhances lung adenocarcinoma metastasis and induces epithelial-mesenchymal transition by sponging miR-375. J BUON. 2019;24:2273–2279.

57. Sarkar A, Rahaman A, Biswas I, Mukherjee G, Chatterjee S, Bhattacharjee S, et al. TGFβ mediated LINC00273 upregulation sponges mir200a-3p and promotes invasion and metastasis by activating ZEB1. J Cell Physiol. 2020;235:7159–7172. doi:10.1002/jcp.29614.

58. Deng JC, Zeng X, Newstead M, Moore TA, Tsai WC, Thannickal VJ, et al. STAT4 is a critical mediator of early innate immune responses against pulmonary Klebsiella infection. J Immunol. 2004;173:4075–4083. doi:10.4049/jimmunol.173.6.4075.

59. Jiang Q, Xue D, Shi F, Qiu J. Prognostic significance of an autophagy-related long non-coding RNA signature in patients with oral and oropharyngeal squamous cell carcinoma. Oncol Lett. 2021;21:29. doi:10.3892/ol.2020.12290.

60. Fang K-Y, Cao W-C, Xie T-A, Lv J, Chen J-X, Cao X-J, et al. Exploration and validation of related hub gene expression during SARS-CoV-2 infection of human bronchial organoids. Hum Genomics. 2021;15:18. doi:10.1186/s40246-021-00316-5.

61. Jain S, Arrais J, Venkatachari NJ, Ayyavoo V, Bar-Joseph Z. Reconstructing the temporal progression of HIV-1 immune response pathways. Bioinformatics. 2016;32:i253–i261. doi:10.1093/bioinformatics/btw254.

62. Badaoui M, Zoso A, Idris T, Bacchetta M, Simonin J, Lemeille S, et al. Vav3 Mediates Pseudomonas aeruginosa Adhesion to the Cystic Fibrosis Airway Epithelium. Cell Rep. 2020;32:107842. doi:10.1016/j.celrep.2020.107842.

63. Dooley HC, Razi M, Polson HEJ, Girardin SE, Wilson MI, Tooze SA. WIPI2 links LC3 conjugation with PI3P, autophagosome formation, and pathogen clearance by recruiting Atg12-5-16L1. Mol Cell. 2014;55:238–252. doi:10.1016/j.molcel.2014.05.021.

64. Su Y-S, Hsieh P-Y, Li J-S, Pao Y-H, Chen C-J, Hwang L-H. The heat shock protein 70 family of chaperones regulates all phases of the enterovirus A71 life cycle. Front Microbiol. 2020;11:1656. doi:10.3389/fmicb.2020.01656.

65. Li G, Zhou L, Zhang C, Shi Y, Dong D, Bai M, et al. Insulin-Like Growth Factor 1 Regulates Acute Inflammatory Lung Injury Mediated by Influenza Virus Infection. Front Microbiol. 2019;10:2541. doi:10.3389/fmicb.2019.02541.

66. Tal MC, Torrez Dulgeroff LB, Myers L, Cham LB, Mayer-Barber KD, Bohrer AC, et al. Upregulation of CD47 is a host checkpoint response to pathogen recognition. MBio. 2020;11. doi:10.1128/mBio.01293-20.

67. Pandey R, Mandal AK, Jha V, Mukerji M. Heat shock factor binding in Alu repeats expands its involvement in stress through an antisense mechanism. Genome Biol. 2011;12:R117. doi:10.1186/gb-2011-12-11-r117.

68. Fitzpatrick T, Huang S. 3’-UTR-located inverted Alu repeats facilitate mRNA translational repression and stress granule accumulation. Nucleus. 2012;3:359–369. doi:10.4161/nucl.20827.

69. Liu WM, Chu WM, Choudary PV, Schmid CW. Cell stress and translational inhibitors transiently increase the abundance of mammalian SINE transcripts. Nucleic Acids Res. 1995;23:1758–1765. doi:10.1093/nar/23.10.1758.

70. Li Y, Jiao J. Deficiency of TRPM2 leads to embryonic neurogenesis defects in hyperthermia. Sci Adv. 2020;6:eaay6350. doi:10.1126/sciadv.aay6350.

71. Zhou Y, Han D. GATA 3 modulates neuronal survival through regulating TRPM 2 in Parkinson ’ s disease. 2017.

72. Miller BA, Cheung JY. TRPM2 protects against tissue damage following oxidative stress and ischaemia-reperfusion. J Physiol (Lond). 2016;594:4181–4191. doi:10.1113/JP270934.

73. Klein B, Destephens A, Dumeny L, Hu Q, He Y, O’Malley K, et al. Hemodynamic influence on smooth muscle cell kinetics and phenotype during early vein graft adaptation. Ann Biomed Eng. 2017;45:644–655. doi:10.1007/s10439-016-1725-0.

74. Watkins-Chow DE, Cooke J, Pidsley R, Edwards A, Slotkin R, Leeds KE, et al. Mutation of the diamond-blackfan anemia gene Rps7 in mouse results in morphological and neuroanatomical phenotypes. PLoS Genet. 2013;9:e1003094. doi:10.1371/journal.pgen.1003094.

75. Wu Y, Zhao T, Deng R, Xia X, Li B, Wang X. A study of differential circRNA and lncRNA expressions in COVID-19-infected peripheral blood. Sci Rep. 2021;11:7991. doi:10.1038/s41598-021-86134-0.

76. Mukherjee S, Banerjee B, Karasik D, Frenkel-Morgenstern M. mRNA-lncRNA Co-Expression Network Analysis Reveals the Role of lncRNAs in Immune Dysfunction during Severe SARS-CoV-2 Infection. Viruses. 2021;13:402. doi:10.3390/v13030402.

77. Vishnubalaji R, Shaath H, Alajez NM. Protein Coding and Long Noncoding RNA (lncRNA) Transcriptional Landscape in SARS-CoV-2 Infected Bronchial Epithelial Cells Highlight a Role for Interferon and Inflammatory Response. Genes (Basel). 2020;11. doi:10.3390/genes11070760.

78. Rodrigues AC, Adamoski D, Genelhould G, Zhen F, Yamaguto GE, Araujo-Souza PS, et al. NEAT1 and MALAT1 are highly expressed in saliva and nasopharyngeal swab samples of COVID-19 patients. Mol Oral Microbiol. 2021;36:291–294. doi:10.1111/omi.12351.

79. Moazzam-Jazi M, Lanjanian H, Maleknia S, Hedayati M, Daneshpour MS. Interplay between SARS-CoV-2 and human long non-coding RNAs. J Cell Mol Med. 2021;25:5823–5827. doi:10.1111/jcmm.16596.

80. Shaath H, Vishnubalaji R, Elkord E, Alajez NM. Single-Cell Transcriptome Analysis Highlights a Role for Neutrophils and Inflammatory Macrophages in the Pathogenesis of Severe COVID-19. Cells. 2020;9. doi:10.3390/cells9112374.

81. Smet A, Breugelmans T, Michiels J, Lamote K, Arras W, De Man JG, et al. A dynamic mucin mRNA signature associates with COVID-19 disease presentation and severity. JCI Insight. 2021.

82. Bose M, Mitra B, Mukherjee P. Mucin signature as a potential tool to predict susceptibility to COVID-19. Physiol Rep. 2020;9:e14701. doi:10.14814/phy2.14701.

83. Guihur A, Rebeaud ME, Fauvet B, Tiwari S, Weiss YG, Goloubinoff P. Moderate Fever Cycles as a Potential Mechanism to Protect the Respiratory System in COVID-19 Patients. Front Med (Lausanne). 2020;7:564170. doi:10.3389/fmed.2020.564170.

84. McCue AD, Nuthikattu S, Reeder SH, Slotkin RK. Gene expression and stress response mediated by the epigenetic regulation of a transposable element small RNA. PLoS Genet. 2012;8:e1002474. doi:10.1371/journal.pgen.1002474.

85. Todd CD, Deniz Ö, Taylor D, Branco MR. Functional evaluation of transposable elements as enhancers in mouse embryonic and trophoblast stem cells. Elife. 2019;8. doi:10.7554/eLife.44344.

86. Bouttier M, Laperriere D, Memari B, Mangiapane J, Fiore A, Mitchell E, et al. Alu repeats as transcriptional regulatory platforms in macrophage responses to M. tuberculosis infection. Nucleic Acids Res. 2016;44:10571–10587. doi:10.1093/nar/gkw782.

87. Jones RB, Song H, Xu Y, Garrison KE, Buzdin AA, Anwar N, et al. LINE-1 retrotransposable element DNA accumulates in HIV-1-infected cells. J Virol. 2013;87:13307–13320. doi:10.1128/JVI.02257-13.

88. Kaneko H, Dridi S, Tarallo V, Gelfand BD, Fowler BJ, Cho WG, et al. DICER1 deficit induces Alu RNA toxicity in age-related macular degeneration. Nature. 2011;471:325–330. doi:10.1038/nature09830.

89. Tarallo V, Hirano Y, Gelfand BD, Dridi S, Kerur N, Kim Y, et al. DICER1 loss and Alu RNA induce age-related macular degeneration via the NLRP3 inflammasome and MyD88. Cell. 2012;149:847–859. doi:10.1016/j.cell.2012.03.036.
