# Supplementary figures and images for: LncRNAs harbouring regulatory motifs within repeat elements modulate immune response towards COVID‐19 disease severity and clinical outcomes
Source: Clin Transl Med. 2022 Jul 8;12(7):e932. doi: 10.1002/ctm2.932 (PMC9270577; doi:10.1002/ctm2.932)

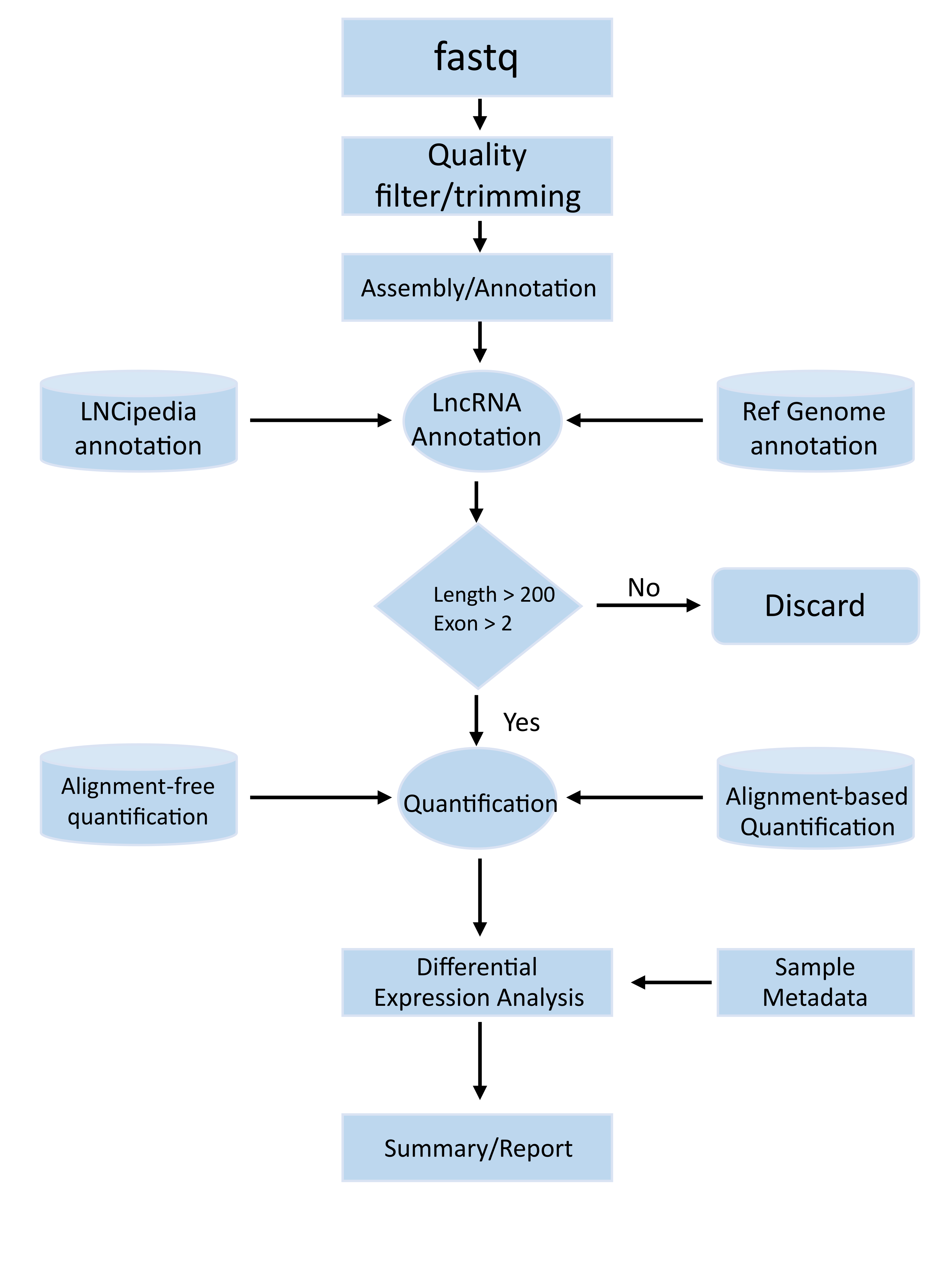

Supplement: Supplementary file 8 — FigureS1 [file CTM2-12-0-s009.png]

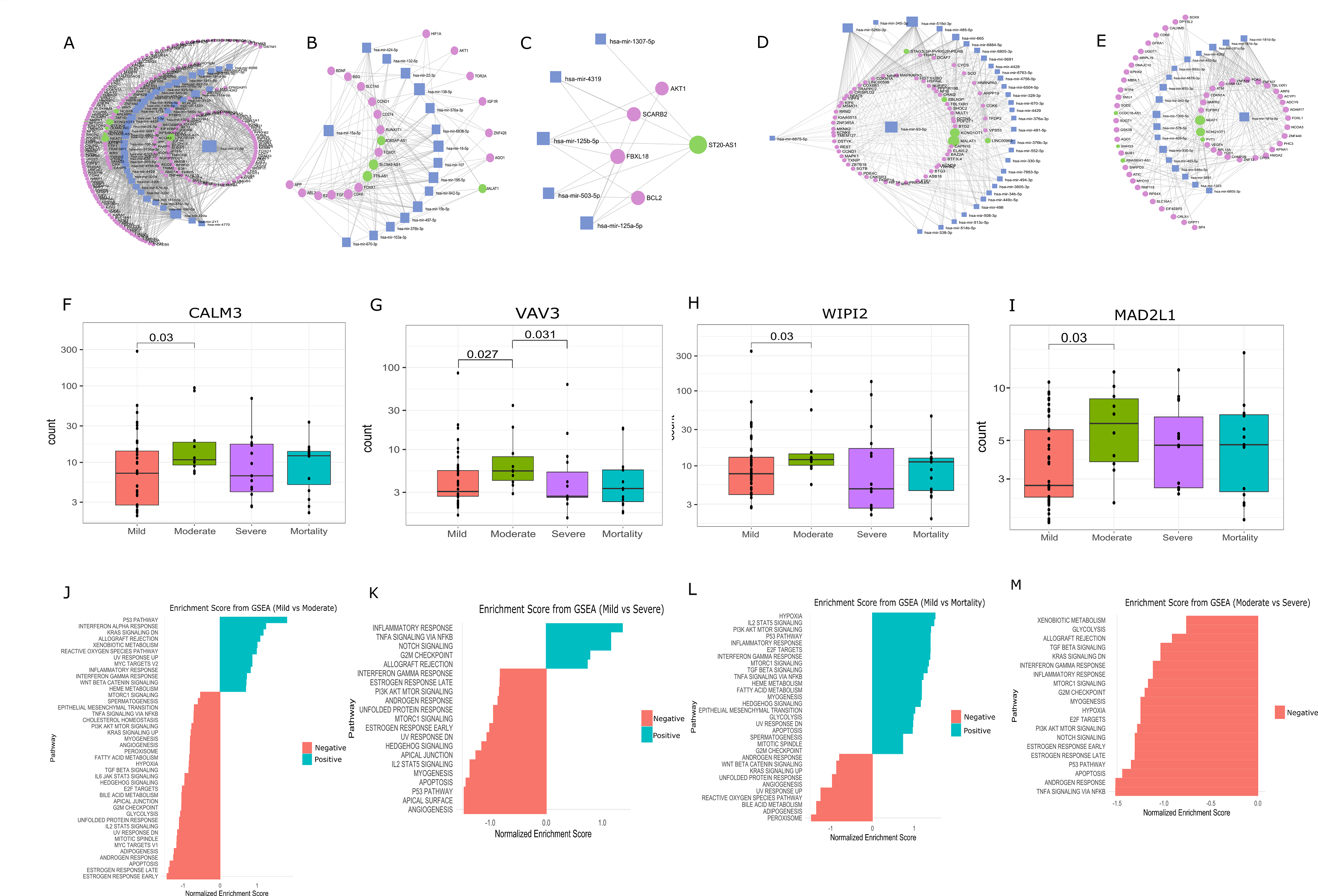

Supplement: Supplementary file 9 — FigureS2 [file CTM2-12-0-s007.png]

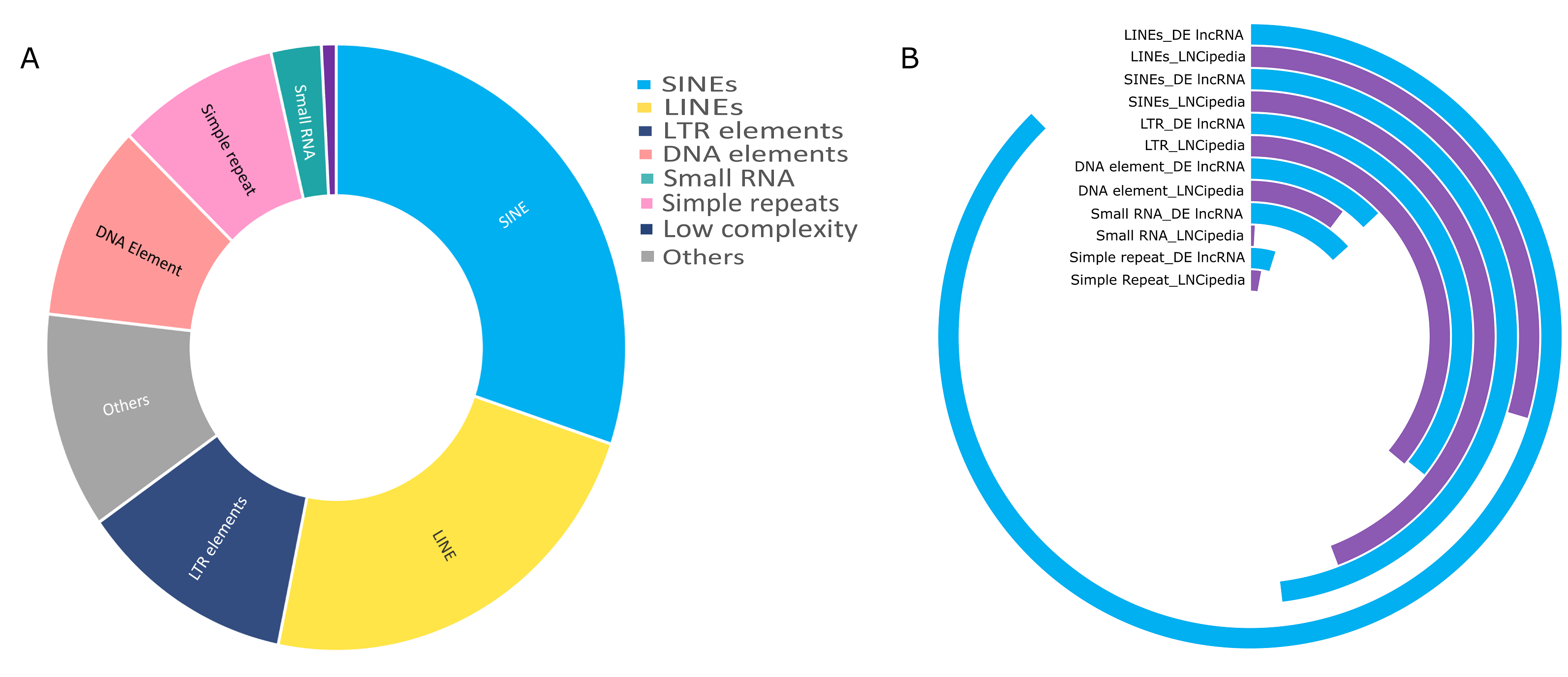

Supplement: Supplementary file 10 — FigureS3 [file CTM2-12-0-s006.png]
